# Dietary compound isoliquiritigenin prevents mammary carcinogenesis by inhibiting breast cancer stem cells through WIF1 demethylation

**DOI:** 10.18632/oncotarget.3396

**Published:** 2015-03-26

**Authors:** Neng Wang, Zhiyu Wang, Yu Wang, Xiaoming Xie, Jiangang Shen, Cheng Peng, Jieshu You, Fu Peng, Hailin Tang, Xinyuan Guan, Jianping Chen

**Affiliations:** ^1^ School of Chinese Medicine, Li Ka Shing Faculty of Medicine, the University of Hong Kong, Hong Kong; ^2^ Department of Mammary Disease, Guangdong Provincial Hospital of Chinese Medicine, The Second Clinical Medical College, Guangzhou University of Chinese Medicine, Guangdong, China; ^3^ Department of Pharmacology, Li Ka Shing Faculty of Medicine, the University of Hong Kong, Hong Kong; ^4^ Department of Breast Oncology, Sun Yat-sen University Cancer Center; State Key Laboratory of Oncology in South China; Collaborative Innovation Center of Cancer Medicine, Guangzhou, China; ^5^ School of Pharmaceutical Science, Chengdu University of Traditional Chinese Medicine, Sichuan, Chengdu, China; ^6^ Department of Clinical Oncology, Li Ka Shing Faculty of Medicine, the University of Hong Kong, Hong Kong

**Keywords:** mammary tumorigenesis, cancer stem cells, WIF1 demethylation, DNMT1, Isoliquiritigenin

## Abstract

Breast cancer stem cells (CSCs) are considered as the root of mammary tumorigenesis. Previous studies have demonstrated that ISL efficiently limited the activities of breast CSCs. However, the cancer prevention activities of ISL and its precise molecular mechanisms remain largely unknown. Here, we report a novel function of ISL as a natural demethylation agent targeting WIF1 to prevent breast cancer. ISL administration suppressed *in vivo* breast cancer initiation and progression, accompanied by reduced CSC-like populations. A global gene expression profile assay further identified WIF1 as the main response gene of ISL treatment, accompanied by the simultaneous downregulation of β-catenin signaling and G0/G1 phase arrest in breast CSCs. In addition, WIF1 inhibition significantly relieved the CSC-limiting effects of ISL and methylation analysis further revealed that ISL enhanced WIF1 gene expression *via* promoting the demethylation of its promoter, which was closely correlated with the inhibition of DNMT1 methyltransferase. Molecular docking analysis finally revealed that ISL could stably dock into the catalytic domain of DNMT1. Taken together, our findings not only provide preclinical evidence to demonstrate the use of ISL as a dietary supplement to inhibit mammary carcinogenesis but also shed novel light on WIF1 as an epigenetic target for breast cancer prevention.

## INTRODUCTION

With the advancements of early-detection systems and multidisciplinary treatments, the 5-year survival ratio of breast cancer has been greatly improved. However, breast cancer incidence has risen sharply since 2008 by more than 20% worldwide, and 1.7 million women were diagnosed with breast cancer in 2012 globally [1]. The development of preventive biomarkers and targeting agents with high safety for breast cancer has become an urgent issue worldwide.

In the past decade, the discovery of CSCs in various types of cancer has challenged our traditional view of cancer incidence. For breast cancer, Al-hajj *et al*. [2] initially identified breast CSCs with CD44^+^/CD24^−/low^ markers in 2003. Breast CSCs were characterized as having strong abilities of self-renewal, tumorigenesis and high metastasis [3]. As few as 100 breast CSCs could form tumors when injected into non-obese diabetic/severe combined immunodeficiency disease (NOD/SCID) mice, and CSCs were also identified to be a critical initiator for the formation of a metastasis lesion [4]. Targeting aberrant signaling pathways in breast CSCs has become a novel approach to effectively prevent breast cancer.

Recent studies indicate that the Wnt/β-catenin signaling pathway plays a critical role in mediating bioactivities of CSCs, including mammosphere formation capability, differentiation potential and tumorigenesis ability [5]. Compared with the Notch and Hedgehog pathways, the Wnt/β-catenin pathway has been strongly implicated in mammary development and tumorigenesis since the discovery of Wnt1 as a mammary oncogene by viral insertion in mouse mammary tumors in the 1980s [6]. In particular, MMTV-Wnt1 transgenic mice display alveolar hyperplasia early in life, and nearly 100% of these animals ultimately develop focal mammary carcinomas [7]. In addition, other Wnt family members including Wnt2, Wnt9a, Wnt7b, and Wnt10b were found to be aberrantly upregulated in human breast cancer tissues when compared with normal mammary tissues [8]. Meanwhile, the high expression of cytoplasmic and/or nuclear β-catenin was found to be closely correlated with breast cancer prognosis [10], and nuclear β-catenin could interact with the lymphocyte enhancer factor/T cell factor family of transcription factors (LEF/TCF) to activate multiple cancer-related molecules, including c-Myc, cyclin D1, metalloproteinases and c-Met, etc. [11–13]. In addition, Wnt/β-catenin signaling could also be abnormally stimulated by oncogenic mutations of several critical components within this pathway, such as APC, Axin and TCF4, etc. [9]. However, mutations in such key regulators were not frequently observed in human breast cancer patients, indicating possible alternative mechanisms for the hyperactivation of the Wnt/β-catenin signaling pathway.

The epigenetic silencing of multiple secreted Wnt antagonists is a strikingly common event in breast cancer, although the causal relationship between their loss-of-expression and mammary carcinogenesis is less well established. There generally exist two classes of secreted Wnt antagonists, defined by their mechanisms of action. The first class encompasses the Dkk family proteins, which internalize the Wnt co-receptors LRP5/6 via endocytosis to suppress downstream signaling transduction. Another class includes Wnt inhibitory factor 1 (WIF1), the sFRP family and Cerberus, which exert inhibitory effects by directly binding to the Wnt ligands [14–15]. Compared to other suppressors, WIF1 inactivation is a common event in breast cancer. Both studies by Ai *et al*. [16] and Veeck *et al.* [17] revealed that WIF1 downregulation by hypermethylation was detected in more than 60% of human breast carcinoma samples. Furthermore, WIF1 is expressed at high levels in human normal breast cells and mammary tissues, suggesting that WIF1 elevation would possibly maintain normal mammary development while attenuating mammary tumorigenesis [18].

Dietary compounds have attracted growing interest for use in cancer chemoprevention and are advantageous owing to their wide availability, low toxicity and high tolerability [19]. Currently, multiple natural dietary compounds are potent in inhibiting breast cancer growth and limiting breast CSCs, such as epigallocatechin-3-gallate (EGCG), resveratrol, and piperine [20–22]. However, their role in breast cancer prevention and the underlying molecular mechanisms remain largely unknown. Isoliquiritigenin (ISL), a chalcone-type dietary compound derived from licorice root and many other plants, possesses anti-cancer activities possibly via proliferation reduction, cell cycle arrest, angiogenesis suppression, metastasis inhibition and apoptosis induction [23–27]. In addition, its chemopreventive role in breast cancer has been reported recently. ISL suppressed phorbol ester-induced cyclooxygenase-2 (COX-2) expression in the non-tumorigenic MCF-10A breast cell line and was capable of inhibiting DMBA-induced mammary carcinogenesis in rats [28–39]. Our pilot results demonstrated that ISL could directly target GRP78 to chemosensitize breast CSCs *via* β-catenin/ABCG2 signaling [13]. Although it was found that ISL limited breast CSCs via increased proteasomal degradation of β-catenin, the effects of ISL on breast cancer chemoprevention and its underlying mechanisms on Wnt/β-catenin signaling deserve further investigation.

In this study, we utilized the mouse mammary tumor virus promoter-driven polyoma middle T oncoprotein (MMTV-PyMT) transgenic mouse model to demonstrate the breast cancer chemoprevention effects of ISL exposure. It was determined that ISL administration suppressed *in vivo* breast cancer initiation and progression and was accompanied by a reduced CSC-like population. Microarray analysis further revealed that WIF1 is the main response gene of ISL, accompanied by limited mammosphere formation ability and G0/G1 phase arrest of breast CSCs. Notably, DNA methylation analysis demonstrated that ISL significantly demethylated the promoter region of WIF1 and inhibited the expression of DNMT1 methyltransferase. Molecular docking analysis revealed that ISL could stably dock into the catalytic domain of DNMT1, thus competitively reversing the WIF1 methylation status. Taken together, our findings discovered a novel function of ISL as a natural DNMT1 inhibitor to prevent breast cancer by targeting the aberrant WIF1 signaling.

## RESULTS

### ISL suppresses mammary hyperplasia and breast cancer initiation *in vivo*

We initially evaluated the chemopreventive effects of ISL on breast cancer in the MMTV-PyMT mice. This mouse model produces spontaneous and luminal-like breast cancer from normal mammary epithelium and can recapitulate similar pathological processes and characteristics found in human breast cancer. In particular, mammary hyperplasia can be detected in this model as early as 4 weeks of age, and nearly 100% of mice develop breast cancer by 12–15 weeks, accompanied by the appearance of pulmonary metastasis [30–31]. Therefore, it is an internationally well-accepted preclinical model for investigating the mechanisms of mammary carcinogenesis and chemopreventive effects of natural phytochemicals.

**Figure 1:**
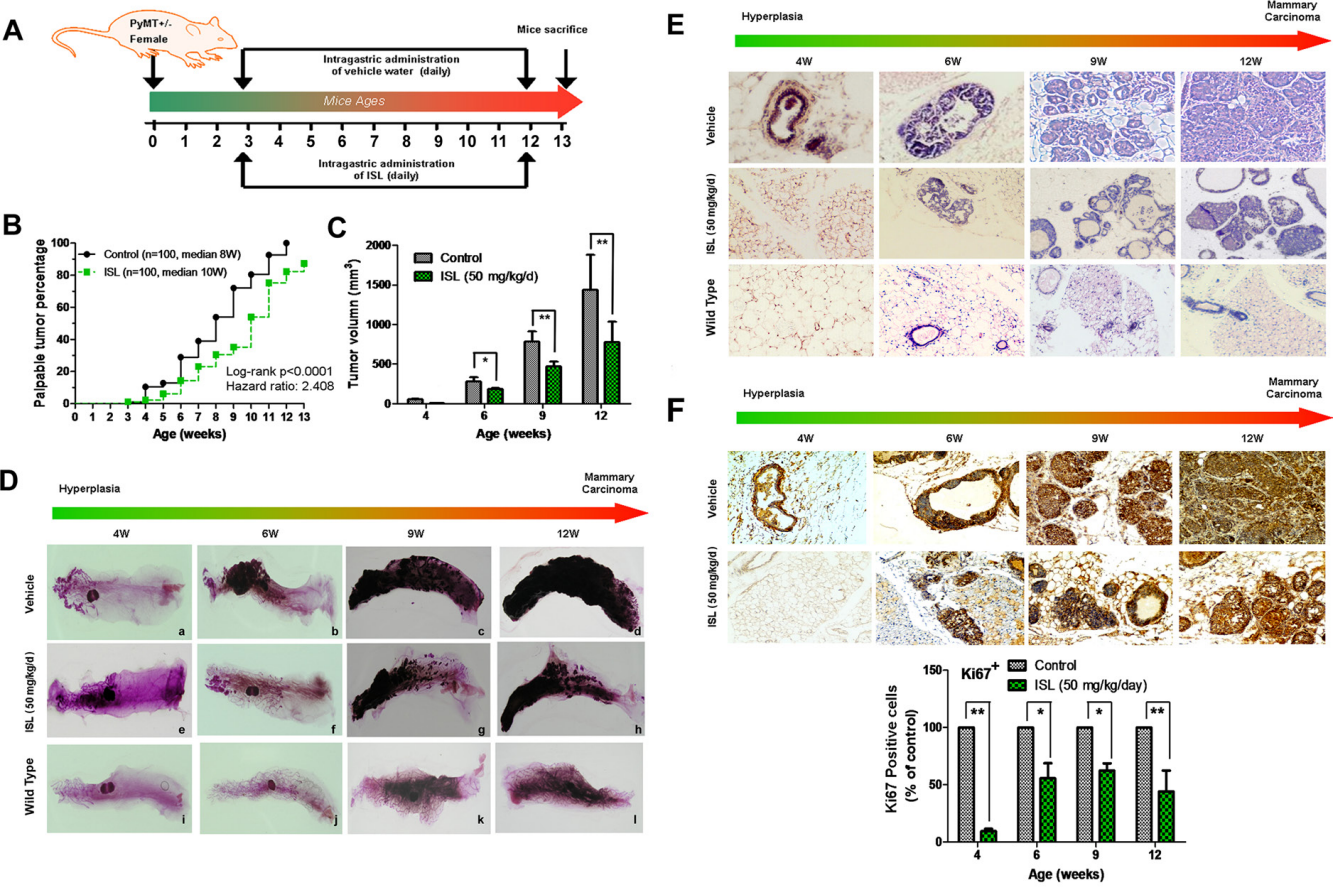
ISL inhibited mammary carcinogenesis in MMTV-PyMT transgenic mice **A.** Schematic illustration of ISL administration. Mice were randomly divided into vehicle and ISL treatment groups, and ISL was given by oral gavage at 50 mg/kg/d from the 3^rd^ to the 12^th^ week (*n* = 10 mice, total of 100 glands); **B.** Tumor incidence ratios between the vehicle and ISL treatment groups were compared using a log-rank test. The results revealed that ISL significantly inhibited breast cancer occurrence (*P* < 0.001); **C.** Tumor volumes of each group were measured from the 4^th^ to the 12^th^ weeks. Tumor volume was calculated based on *ex vivo* caliper measurements of individual tumors using the formula *V* = (*W*^2^× *L*)/2, where V is tumor volume, W is tumor width, and L is tumor length. The results indicated that ISL significantly inhibited tumor growth (*P < 0.05, **P < 0.01, values represented as the Mean ± SD, *n* = 10). **D.** Whole mount staining of abdominal mammary glands of mice from the 4^th^ to 12^th^ weeks in the wild type, vehicle, and ISL intervention groups; **E.** Representative H&E staining images of mouse mammary glands from the 4^th^ to the 12^th^ weeks in the wild type, vehicle, and ISL intervention groups. **F.** Representative immunohistochemical images of Ki 67 staining in mouse mammary glands from the 4^th^ to the 12^th^ weeks in the wild type, vehicle, and ISL intervention groups.

ISL (50 mg/kg/d) and vehicle were administered to mice by oral gavage everyday according to the schedule illustrated in Figure 1A. The cancer prevention activity of ISL was initially assessed by comparing the incidence of palpable lesions at different time intervals in the transgenic mice of each group. As shown in Figure 1B, ISL treatment obviously delayed the onset of palpable lesions. The cancer progression was also significantly slowed down by ISL intervention when compared with the vehicle group. In particular, the median time to a 50% cancer-burden in the glands was 8 weeks in the vehicle group, while it was 10 weeks in the ISL-treated group. By the 12^th^ week, all mammary glands (*n* = 100) were identified as breast cancer in the vehicle group, while more than 10 glands were free of lesions in the ISL treatment group. Meanwhile, the mean tumor volume from the 4^th^ to 12^th^ weeks in the ISL intervention group was also significantly decreased compared with the vehicle group, as presented in Figure 1C.

To further investigate the effects of ISL on breast tumor initiation, the abdominal mammary glands of mice were harvested for whole mount preparation at varying stages. At 4 weeks after birth, only a rudimental primary duct and a small number of branches can be observed in wild-type mice (Figure 1Di). As the mice age, the length of ducts, the amount of duct branches, and the number of terminal ends gradually grow in wild-type mice (Figure 1Dj–l). Alternatively, in MMTV-PyMT transgenic mice, hyperplastic lesions could be observed as early as the 4^th^ week around duct branches, while no lesions were detected in the ISL treatment group at the same age (Figure 1De). As the mice grew older, the transgenic mice gave rise to increasing numbers of mammary tumors, while ISL treatment significantly limited tumor foci growth and dispersion (Figure 1Dc,g).

Hematoxylin and eosin (H&E) staining further showed the effects of ISL on the cellular morphology of mouse mammary glands at varying stages. As shown in Figure 1E, the 4-week-old vehicle group began to develop hyperplastic lesions, which presented as multiple foci around the ducts. The cellular morphology of an ISL-treated gland was similar to that of wild type, and almost no premalignant lesions were found. In the 6^th^ week of age, hyperplastic lesions were present in the mammary glands of the control and ISL-treated groups. However, ISL treatment markedly decreased the malignant cell proliferation within the acini and ducts when compared with the control group in the 9^th^ week. With tumor progression to the 12^th^ week, individual tumor acini began to merge in the control group, while no such feature was found in the ISL-treated group. Immunohistochemical analysis also showed that ISL treatment could decrease Ki67 expression in the tumor tissues at varying stages, indicating the proliferation speed of tumor cells was remarkably inhibited by ISL (Figure 1F). Collectively, ISL significantly prevented mammary cancer occurrence and growth in the MMTV-PyMT transgenic mouse model.

### ISL treatment inhibits breast cancer growth and lung metastasis *in vivo*

**Figure 2:**
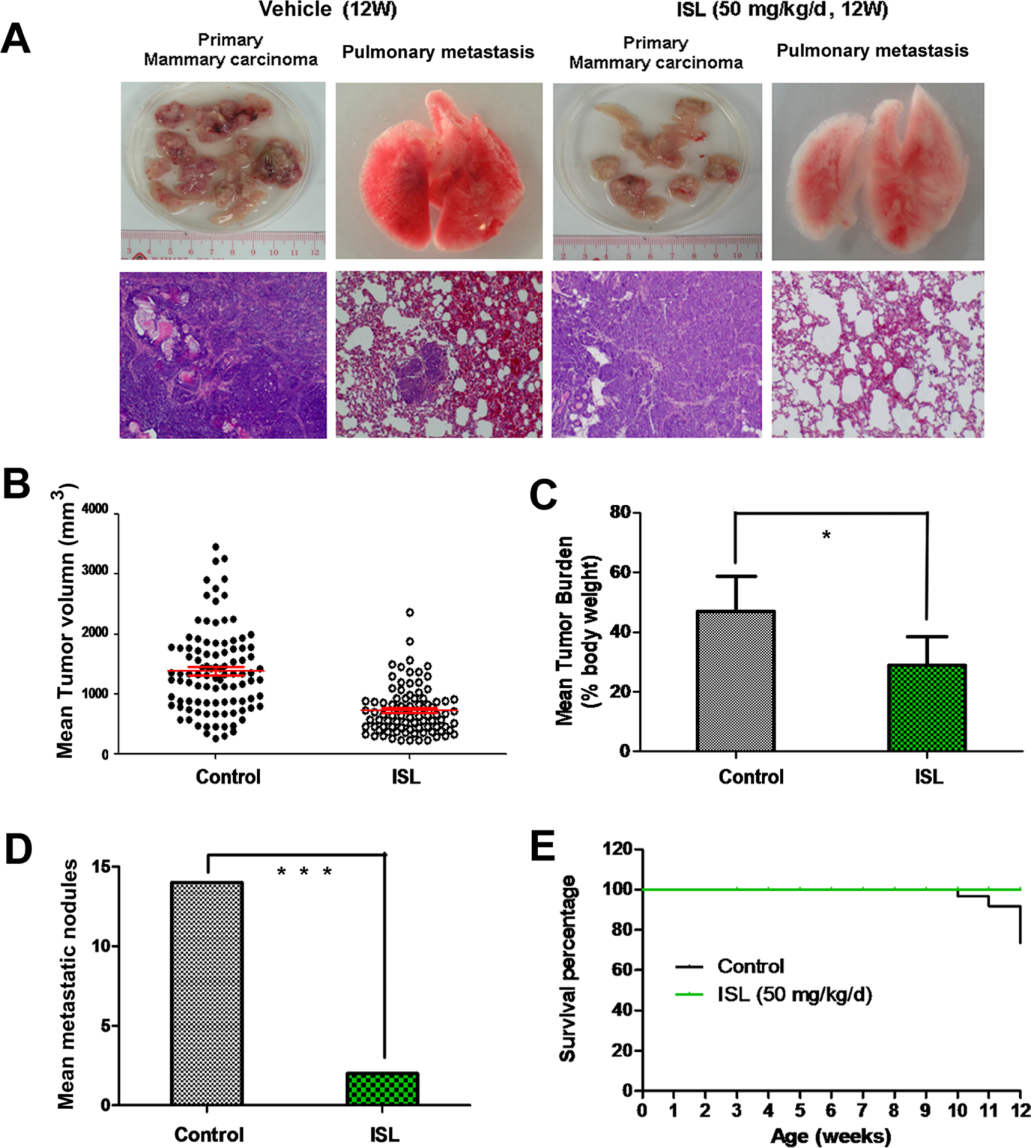
ISL inhibited breast cancer growth and lung metastasis in MMTV-PyMT transgenic mice **A.** Representative images of tumors and lungs dissected from vehicle or ISL-treated mice. H&E staining was utilized to observe tumor micromorphology of each group with 400 fold magnification; **B.** Scatter plot of individual tumors and the mean tumor volume (red line) and the SEM derived from mice tumors at the end of ISL treatment (50 mg/kg/d); **C.** Mean tumor burden per mouse at the experimental endpoint, analyzed by Student's *t* test. Tumor burden per mouse was calculated as tumor weight per body weight (*P < 0.05, values represented as the mean ± SD, *n* = 10); **D.** Mean metastatic nodule count in each group at the experimental endpoint was compared (***P < 0.001, values represented as the mean ± SD, *n* = 10); **E.** Mouse survival in the vehicle and ISL intervention groups is shown by the Kaplan-Meier curve.

Accumulating evidence has suggested that MMTV-PyMT mice could develop well-differentiated, luminal-type mammary adenomas with lung metastasis lesions in or after the 10^th^ week [32]. Therefore, we further examined the effects of ISL on breast cancer growth and metastasis at the 13^th^ week, the endpoint of our experiment. As shown in Figure 2A, mammary tumors from the vehicle group displayed a more necrotic and hemorrhagic appearance than ISL-treated tumors, while no obvious visual differences in the pulmonary specimens were observed between the two groups. H&E analysis further indicated that tumors from both groups showed almost the same features of malignancy, including solid sheets of malignant cells, marked cell morphology variation, obvious nuclear atypia and the loss of the basement membrane. Meanwhile, several metastatic nodules were present in the lung parenchyma of the control group, while few such lesions were visible in the ISL-treated group. Although ISL treatment did not result in obvious changes in the histopathology of the end-stage tumors based on H&E staining, it significantly inhibited the tumor volume and tumor burden. ISL elicited a dramatic decrease of 658 mm^3^ in the mean tumor volume, and a significant 18% reduction in the tumor burden compared to the vehicle groups (Figure 2B, 2C). The discrepancy in the mean number of metastatic nodules further revealed that ISL harbored the potential for limiting metastasis (Figure 2D). Kaplan-Meier curve analysis further demonstrated that ISL significantly prolonged the survival time of MMTV-PyMT mice, possibly owing to the decreased tumor burden and the limited lung metastasis (Figure 2E). Additionally, ISL had little effect on mouse body weight between the two groups, indicating that ISL might be developed as a chemoprevention agent with high safety (Supplementary Figure 1).

### Identification of WIF1 by microarray profiling as the primary target of ISL in limiting breast CSC

**Figure 3:**
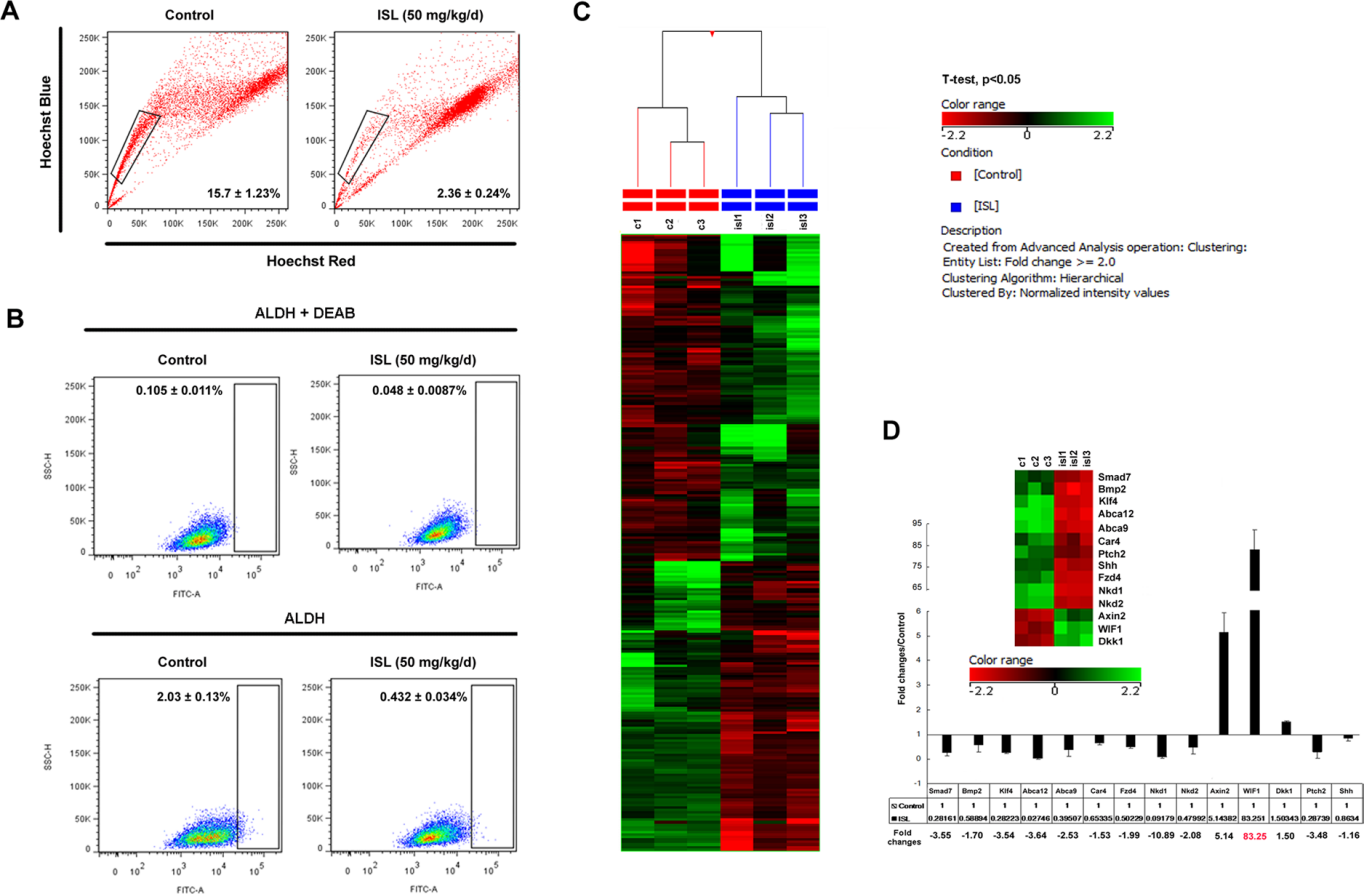
Identification of WIF1 as the main target of ISL by microarray profiling **A.** Representative SP analysis using primary mouse mammary cells freshly harvested from the spontaneous mammary tumors of vehicle or ISL-treated MMTV-PyMT mice. The SP (the framed area) was shown as a percentage of the viable cell population and analyzed by FlowJo software; **B.** The ALDEFLUOR assay was then used to determine the population of CSCs in the spontaneous mammary tumors of vehicle or ISL-treated MMTV-PyMT mice. An ALDH-specific inhibitor DEAB was used as a negative control for minimizing background fluorescence. ALDH^hi^ cells were shown as cells residing in the framed area analyzed by FlowJo software; **C.** Affymetrix Mouse Gene 2.0 ST GeneChip was utilized to reveal the gene expression changes after ISL treatment in MMTV-PyMT mice. Through GeneSpring12.6 analysis, 132 genes were upregulated, whereas 117 genes were downregulated; **D.** 14 genes including Smad7, Bmp2, Klf4, Abca12, Abca9, Ca4, Fzd4, Nkd1, Nkd2, Ptch2, Shh, Axin2, WIF1 and Dkk1 were identified as CSC-related genes affected by ISL. Real-time PCR analysis was then applied to validate their expression changes and WIF1 was finally determined as the main response gene of ISL.

Accumulating evidence has suggested that cancer is a stem cell disorder. These rare CSCs are possibly the ultimate roots of tumorigenesis, tumor recurrence and metastasis [33]. To investigate the effects of ISL on the origination of breast CSCs, we harvested primary cells from the spontaneous mammary tumors of MMTV-PyMT mice for flow cytometry analysis. Two independent markers for breast CSCs were applied. First, a Hoechst 33342-based “side population” (SP) assay was utilized for detecting the effects of ISL on the CSC population. ISL treatment reduced the cancer stem-like cell proportion from 15.7 ± 1.23 to 2.36 ± 0.24 (Figure 3A). Aldehyde dehydrogenase (ALDH) was then applied as another marker for detection of stem-like cells and a specific inhibitor diethylaminobenzaldehude (DEAB) against ALDH was used as a negative control to minimize the influence of background fluorescence [34]. Compared with the DEAB negative control, tumors from untreated mice consisted of approximately 2% ALDH^hi^ cells, while ISL administration dramatically decreased ALDH^hi^ cells to 0.4% (Figure 3B). Both results indicated that ISL might prevent breast cancer occurrence and development via limiting breast CSCs.

Microarray analysis was then applied to identify genes that could be affected by ISL. The candidate gene profiles of MMTV-PyMT tumors with or without ISL were determined by identification of genes with expression changes at least 2.0-fold and with a *P* value less than 0.05. As indicated in Figure 3C, a total number of 249 genes were finally selected, among which 132 genes were up-regulated, and 117 genes were down-regulated. Meanwhile, 14 genes were found that were involved in CSC activities and categorized into functional clusters that included Wnt/β-catenin signaling, Hedgehog signaling and other CSC-related genes. Then, a real-time PCR assay was used to validate the results of the microarray analysis. As shown in the right panel of Figure 3D, the decreased expression of Smad7, Bmp2, Klf4, Abca12, Abca9, Car4, Fzd4, Nkd1, Nkd2, Ptch2 and Shh and the up-regulation of Axin2, WIF1 and Dkk1 were all validated. Meanwhile, it was revealed that WIF1 was the gene with the highest expression alteration by ISL among the 14 candidate genes (Figure 3D).

**Figure 4:**
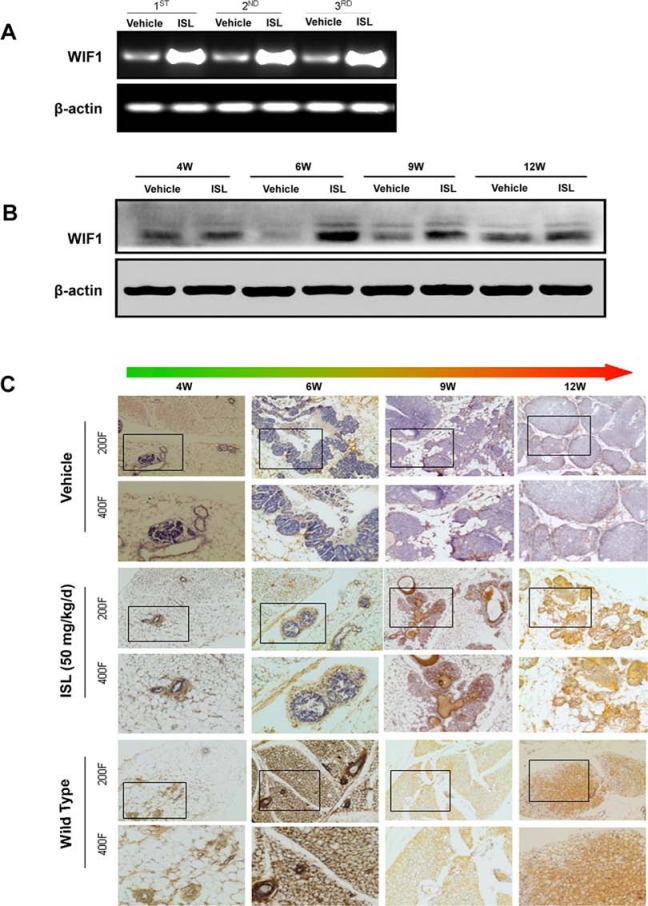
ISL significantly elevated WIF1 expression in MMTV-PyMT mice **A.** Real-time PCR analysis revealed that ISL significantly increased WIF1 mRNA expression in mammary tumors of MMTV-PyMT mice at the end of the experiment; **B–C.** Western blotting and immunohistochemistry analysis further confirmed that ISL gradually increased WIF1 expression from the 4^th^ to the 12^th^ week.

To further examine the relevance of WIF1 to the chemopreventive effects of ISL, the mRNA levels of WIF1 in mammary tissues from vehicle and ISL-treated groups were examined by RT-PCR. The results revealed a significant elevation of WIF1 by ISL treatment when compared to the vehicle control (Figure 4A). In addition, western blot analysis also confirmed that ISL administration led to an increase in WIF1 expression levels as time progressed from the 4^th^ to the 12^th^ week (Figure 4B). Furthermore, immunohistochemistry staining showed that WIF1 was expressed in wild-type mammary tissues in mice from weanlings to adults (Figure 4C). However, WIF1 expression was significantly attenuated in MMTV-PyMT mice, particularly after tumor development. Notably, augmentation of WIF1 expression was observed in ISL-treated mammary tissues, especially when the hyperplasia had developed to the carcinoma stage at the 12^th^ week of age. Additionally, the distribution of WIF1 positive cells at the hyperplasic stage (4–6 weeks) was quite different from that at the carcinoma stage (12 weeks); specifically, WIF1 localization changed from primarily the outer layer of lesions to being distributed throughout the entire tumor.

### ISL limits the self-renewal ability of human breast CSCs *in vitro*

**Figure 5:**
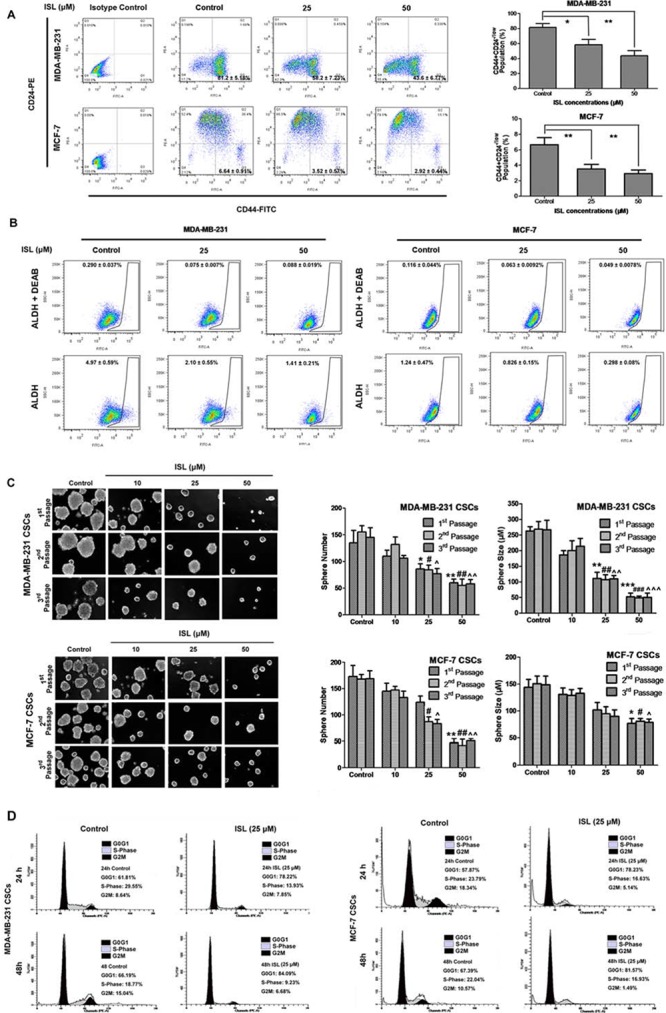
ISL limited the self-renewal ability of breast CSCs **A.** Representative dot plots of CD44^+^CD24^−/low^ cell surface markers in MDA-MB-231 and MCF-7 cells using the BD FACSAria SORP cell sorter. Breast cancer cells were incubated with 25 or 50 μM ISL for 24 h. CD44-FITC and CD24-PE antibodies were utilized to frame CSC-like subsets in the lower right quadrant (Q3). The isotype-matched antibody served as a negative control. The data analysis was performed using FlowJo software (*P < 0.05, **P < 0.01, values represented as the mean ± SD, *n* = 3). The results suggested that ISL administration significantly reduced the CSCs population in both cancer cells in a dose-dependent manner; **B.** The ALDEFLUOR assay was then used to determine the population of CSCs in MDA-MB-231 and MCF-7 cells after ISL administration. The ALDH-specific inhibitor DEAB was used as a negative control for minimizing background fluorescence. ALDH^hi^ cells were shown as cells residing in the framed area analyzed by FlowJo software; time-dependent manner in both CSCs. **C.** Effects of ISL on the primary, secondary and tertiary mammospheres formed by the sorted CSCs from MDA-MB-231 and MCF-7 cells. The CSCs were incubated with or without ISL (10, 25 or 50 μM) for 7 days. The number and size of the mammospheres were determined using fluorescence microscopy (*P < 0.05, **P < 0.01, ***P < 0.001 *vs*. negative control of the 1^st^ passage spheres; *P < 0.05, **P < 0.01, ***P < 0.001 *vs*. negative control of the 2^nd^ passage spheres; ^*P* < 0.05, ^^*P* < 0.01, ^^^*P* < 0.001 *vs*. negative control of the 3^rd^ passage spheres, values represented as the mean ± SD, *n* = 3); the results showed that ISL administration could significantly limit the number and size of mammospheres formed by both CSCs populations; **D.** The effects of ISL on cell cycle distributions of the sorted CSCs from MDA-MB-231 and MCF-7 cells. The CSCs were incubated with or without 25 μM ISL at 24 h and 48 h and then subjected to cell cycle investigation analyzed by Modifit LT software. The results indicated that ISL arrested cell cycle at G0/G1 phase in a time-dependent manner in both CSCs.

After *in vivo* examination, the classic “stem-like” phenotype of CD44^+^CD24^−/low^ was applied to determine the effects of ISL on the MDA-MB-231 triple-negative breast cancer cell line and the MCF-7 luminal cancer cell line. Flow cytometry results showed that ISL could significantly limit the population of breast CSCs in both cell lines (Figure 5A). In particular, 25 μM ISL eliminated the CD44^+^CD24^−/low^ population of MDA-MB-231 cells by 23%, whereas 50 μM ISL resulted in over a 37% reduction of CSC-like cells. Similar findings were also obtained in MCF-7 cells. Compared to 6.64% of breast CSCs in the untreated MCF-7 cells, the proportion of CD44^+^CD24^−/low^ cells dropped to 3.52% and 2.92% after treatment with 25 and 50 μM ISL for 24 h, respectively. ALDH was also applied for detection of stem-like cells with or without ISL plus the specific inhibitor DEAB. It was shown that ISL at 50 μM could reduce ALDH factions from 4.97% to 1.41% in MDA-MB-231 and from 1.24% to 0.298% in MCF-7, indicating that ISL could suppress the ALDH^hi^ population in both cell lines (Figure 5B). The results suggested ISL could decrease CSC populations characterized with either ALDH^hi^ or CD44+CD24^−/low^ markers.

To study the effects of ISL on the self-renewal ability of breast CSCs, we further sorted breast CSCs from MDA-MB-231 and MCF-7 cells and performed a mammosphere formation assay. As shown in Figure 5C, untreated MDA-MB-231 CSCs could be enriched in non-adherent spherical clusters of cells. However, ISL treatment significantly inhibited the formation of primary spheres. Compared to the control group, the number of spheres at 1^st^ passage declined to 63.7% and the size of mammospheres was also inhibited to 42.2% after addition of 25 μM ISL. Additionally, the limited self-renewal ability of ISL-treated cells was also evident in the secondary and tertiary propagation of breast CSCs. Similar inhibitory effects of ISL were observed in the breast CSCs that were sorted from MCF-7 cells.

Because it was reported that WIF1 expression might be correlated with cell cycle progression, we studied the effect of ISL on the cell cycle distribution of breast CSCs. In the sorted MDA-MB-231 CSCs, 25 μM ISL could arrest cell cycle at the G0/G1 phase after 24 h (78.22% of ISL-treated cells versus 61.81% of untreated cells) and 48 h treatment (84.09% of ISL-treated cells versus 66.19% of untreated cells). Similar inhibitory activities of ISL on the G0/G1 population were obtained in the MCF-7 CSCs. The proportion of ISL-treated CSCs in G0/G1 phase increased from 57.87% to 78.23% after 24 h, and continued to elevate from 67.39% to 81.57% after 48 h. These results suggested that ISL might limit the self-renewal ability of breast CSCs via cell cycle arrest at G0/G1 phase (Figure 5D).

### ISL inhibits Wnt/β-catenin signaling in a WIF1 dependent manner

**Figure 6:**
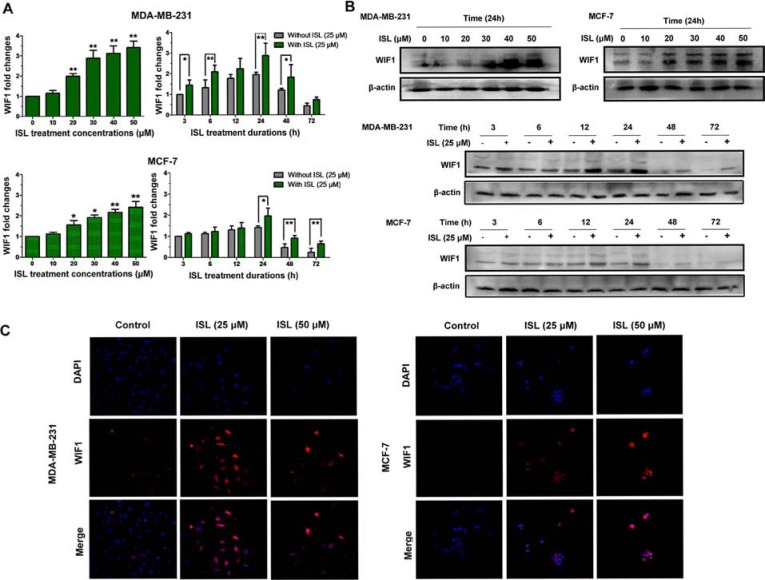
ISL resulted in overexpression of WIF1 levels in human breast cancer cell lines Breast cancer cells were treated with the indicated concentrations of ISL for different times and collected for **A.** Real-time PCR and **B.** western blotting analysis. The results indicated that following ISL treatment, WIF1 expression in both cancer cell lines reached the maximum level at 24 h and then began to decrease with time increasing; **C.** The distribution of WIF1 in MDA-MB-231 and MCF-7 cells after ISL treatment was studied by immunofluorescence analysis. The nuclei were counterstained by DAPI. Fluorescent images were obtained using a Carl Zeiss LSM710 META laser scanning confocal microscope and were analyzed with ZEN software.

After validating the effects of ISL on limiting breast CSCs, we continued to examine whether ISL could exert stimulatory effects on WIF1 in breast cancer cell lines. As illustrated in Figure 6A, both breast cancer cell lines treated with ISL showed dose-dependent elevations of WIF1 transcript levels. However, it was observed that WIF1 mRNA expression reached the highest point at 24 h of treatment and then gradually decreased after prolonged culture in both cell populations, suggesting that the WIF1 gene expression might be correlated with cell cycle progression, consistent with the findings of Liu *et al* [35]. To further evaluate the endogenous protein levels of WIF1 in the presence or absence of ISL, WIF1 expression was further examined by western blot (Figure 6B) and immunofluorescent staining (Figure 6C). Both results revealed that ISL could stimulate WIF1 expression in both breast cancer cell lines.

**Figure 7:**
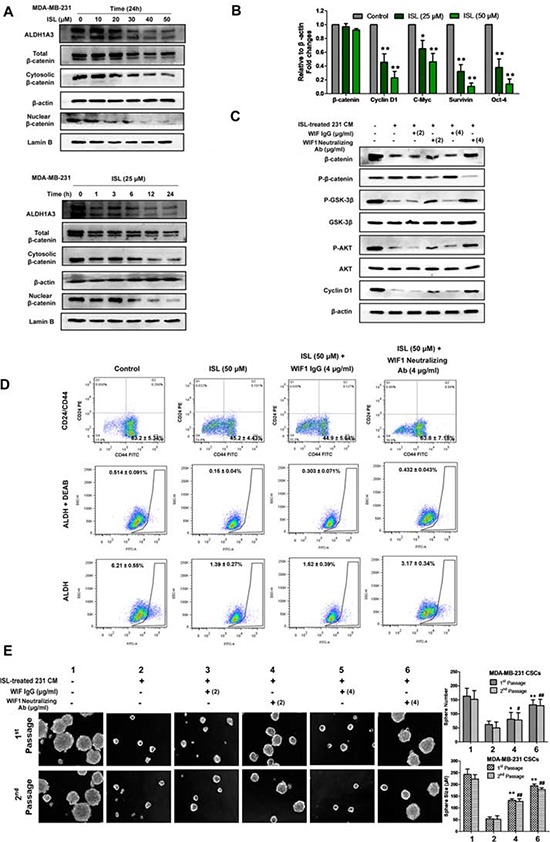
ISL inhibited breast CSCs in a WIF1-dependent manner **A.** MDA-MB-231 cells treated with ISL at varying concentrations or time intervals were assayed by western blotting for β-catenin (cytoplasmic and nuclear) and ALDH1A3 antigen. β-actin and Lamin B were used as cytoplasmic and nuclear protein loading controls, respectively. The results indicated that ISL administration inhibits ALDH1A3 and β-catenin expression in a dose-and time-dependent manner; **B.** The relative β-catenin mRNA levels and the transcriptional activities of its downstream genes before and after ISL treatment in MDA-MB-231 were determined by real-time PCR analysis. (*P < 0.05, **P < 0.01, ***P < 0.001, values represented as the mean ± SD, *n* = 3); **C.** Breast CSCs of MDA-MB-231 were cultured in ISL-treated CM, and 2 or 4 μg/ml WIF1 neutralizing antibody was added to the culture system. Western blotting results revealed that WIF1 inhibition relieved the β-catenin-inhibitory effects of ISL, accompanied by the reactivation of Cyclin D1 and the phosphorylation of GSK-3β and AKT; **D.** WIF1 inhibition reversed the inhibitory effects of ISL on CSC populations in MDA-MB-231 cells; **E.** WIF1 inhibition abrogated the inhibitory effects of ISL on the mammosphere formation ability of CSCs. Breast CSCs were cultured in ISL-treated CM, and WIF1 neutralizing antibody was added to the culture system at 2 or 4 μg/ml. The number and size of the primary and secondary mammospheres were determined using fluorescence microscopy after 7 days (*P < 0.05, **P < 0.01 *vs*. negative control of the 1^st^ passage spheres, *P < 0.05, **P < 0.01 *vs*. negative control of the 2^nd^ passage spheres, values represented as the mean ± SD, *n* = 3).

Because Wnt/β-catenin signaling is closely correlated with the self-renewal ability of breast CSCs, we therefore determined the inhibitory effects of ISL on β-catenin expression in MDA-MB-231 cells. As shown in Figure 7A, ISL inhibited both cytosolic and nuclear β-catenin expression in a time- and dose-dependent manner but without great influences on its mRNA expression. Meanwhile, a series of genes downstream of β-catenin, including Cyclin D1, C-Myc, Survivin and Oct-4, were also simultaneously suppressed by ISL, indicating that the down-regulation of β-catenin might occur at the post-translational level (Figure 7B). To elucidate whether the enhanced WIF1 expression is critical for inducing the down-regulated β-catenin expression by ISL, we cultured breast CSCs in ISL-treated conditional medium (CM) of MDA-MB-231 cells, and a WIF1 neutralizing antibody was added to the culture system to see whether WIF1 inhibition would relieve the CSC-limiting effects of ISL. Western blotting results showed that when compared to the negative control, ISL CM significantly inhibited β-catenin expression *via* phosphorylating β-catenin and dephosphorylating GSK-3β. However, the expression of β-catenin was elevated by 24% and 65% after adding WIF1 neutralizing antibody to the culture system at 2 μg/ml and 4 μg/ml, respectively (Figure 7C). Meanwhile, flow cytometry results also demonstrated that WIF1 inhibition could reverse the down-regulation of breast CSCs induced by ISL in MDA-MB-231 breast cancer cells, but an isotype-matched IgG at 4 μg/ml did not (Figure 7D). On the other hand, mammosphere results showed that ISL CM could significantly limit the number and size of the mammospheres compared to the negative control, while the number and size of the primary mammospheres were increased to 1.3- and 2.4-fold, respectively, by adding anti-WIF1 antibody at 2 μg/ml, and to 2.1- and 3.6-fold, respectively, at 4 μg/ml, but not by an isotype-matched IgG (Figure 7E). These results indicated that ISL inhibited the Wnt/β-catenin signaling in a WIF1-dependent manner.

### ISL elevates WIF1 expression by promoter demethylation through inhibiting DNMT1

**Figure 8:**
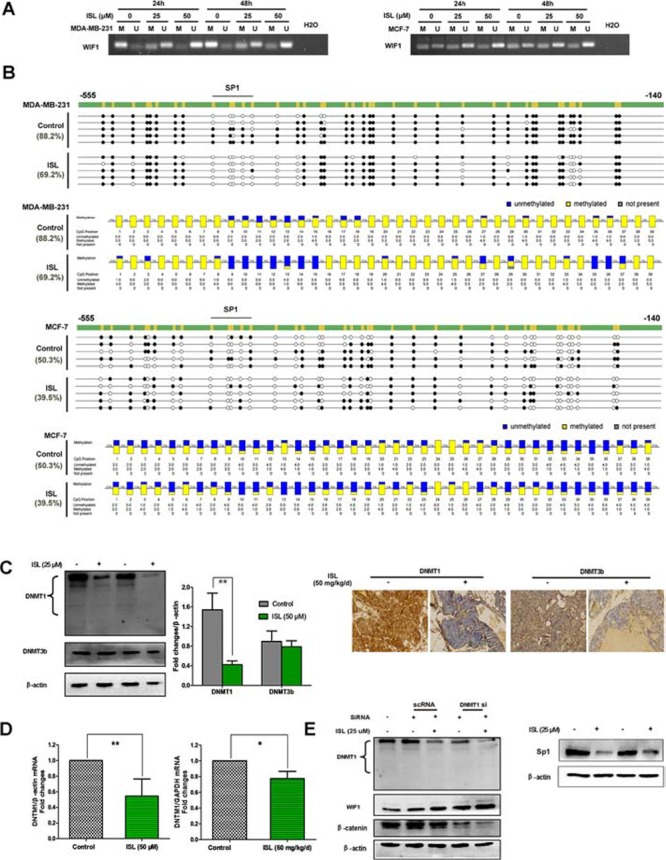
ISL elevated WIF1 expression by promoter demethylation through inhibiting DNMT1 expression **A.** MSP analysis was conducted on bisulfite-modified genomic DNA harvested from MDA-MB-231 and MCF-7 in the presence or absence of ISL at varying concentrations. The results indicated that ISL significantly increased the expression of unmethylated WIF1 promoter DNA fragments; **B.** BGS analysis further confirmed that ISL significantly demethylated the promoter region of WIF1 in both breast cancer cells. For BGS lollipop-style representation, filled (black) circles correspond to methylated Cs, and unfilled (white) circles correspond to unmethylated Cs. Each row represents the sequencing results from a single clone. For BGS aggregated representation, each box corresponds to one CpG position in the genomic sequence. The colored bars summarize the methylation states of all sequences at that position; **C.** Western blotting and immunohistochemistry analysis indicated that ISL significantly inhibited the level of DNMT1 expression rather than DNMT3b; **D.** DNMT1 mRNA levels in MDA-MB-231 cells and tumor tissues before and after ISL treatment were assessed by RT-PCR, and normalized to β-actin or GAPDH internal control. (*P < 0.05, **P < 0.01, values represented as the mean ± SD, *n* = 3); **E.** *Left panel*: DNMT1 siRNA administration further enhanced the stimulatory effects of ISL on WIF1 expression, indicating the critical role of DNMT1 in mediating the demethylation effects of ISL; *Right panel*: ISL inhibited the expression of SP1.

Accumulating evidence has supported that low levels of WIF1 in breast cancer were largely attributed to its aberrant hypermethylation [16]. To further unravel the potential mechanisms underlying the stimulatory effects of ISL on WIF1 expression, we next investigated whether ISL would suppress WIF1 promoter methylation in breast cancer cells. An MSP primer set (WIF1 MSP-F and WIF1 MSP-R) and BGS primer set (WIF1 BGS-F and WIF1 BGS-R) were designed to target the WIF1 promoter region. When MSP analysis was used on bisulfite-modified genomic DNA harvested from the untreated MDA-MB-231 and MCF-7 cells, we observed strong amplification with the methylation-specific primers, indicating a high CpG methylation status within the WIF1 promoter. In contrast, ISL administration led to WIF1 promoter demethylation in a dose- and time-dependent manner, presenting as decreased amplification with methylation-specific primers and increased amplification with unmethylated-specific primers (Figure 8A).

To confirm our MSP results and determine how ISL affects WIF1 promoter methylation in breast cancer, we used bisulfite sequencing to analyze the methylation status of 39 CpG sites within the 415-bp fragment of the WIF1 promoter (from −555 to −140) in MDA-MB-231 and MCF-7 cells (Figure 8B). Consistent with the MSP results, we found that these CpG islands were densely methylated in the detected sequences of both of the untreated cell lines, presenting as 88.2% methylated in MDA-MB-231 and 50.3% methylated in MCF-7 cells. After ISL treatment, the methylation levels were significantly reduced (69.2% methylation in ISL-treated MDA-MB-231 cells, 39.5% methylation in ISL-treated MCF-7 cells). These results suggested that the enhanced WIF1 expression by ISL might largely correlate with reduced promoter methylation of this gene during breast tumorigenesis.

Elevated expression of DNA methyltransferases (DNMTs), mainly DNMT1 and DNMT3B, has been reported to be largely responsible for the aberrant WIF1 hypermethylation in breast tumors [16]. We thus continued to assess whether ISL could suppress the DNMTs to mediate the WIF1 demethylation effects of ISL. Both *in vitro* western blotting and *in vivo* IHC analysis revealed that ISL had little influence on DNMT3b, while it significantly inhibited DNMT1 protein expression (Figure 8C). These results suggested that the demethylation activity of ISL on WIF1 was mainly through the inhibition of DNMT1 rather than DNMT3b. The mRNA levels of DNMT1 were also decreased after ISL administration *in vitro* and *in vivo* (Figure 8D). In addition, DNMT1 silencing could assist ISL not only in elevating WIF1 expression but also in down-regulating β-catenin levels, implying that DNMT1 played a crucial role in mediating the chemoprevention effects of ISL (*Left panel*, Figure 8E). The specificity protein 1 (Sp1) is a zinc finger transcription factor that preferentially binds to GC-rich motifs of many promoters and is capable of transcriptionally regulating DNMT1 [35]. Herein, we found that ISL administration led to a significant reduction of Sp1 levels (*Right panel*, Figure 8E), indicating that ISL promoted WIF1 demethylation possibly through inactivating a Sp1/DNMT1-dependant pathway.

**Figure 9:**
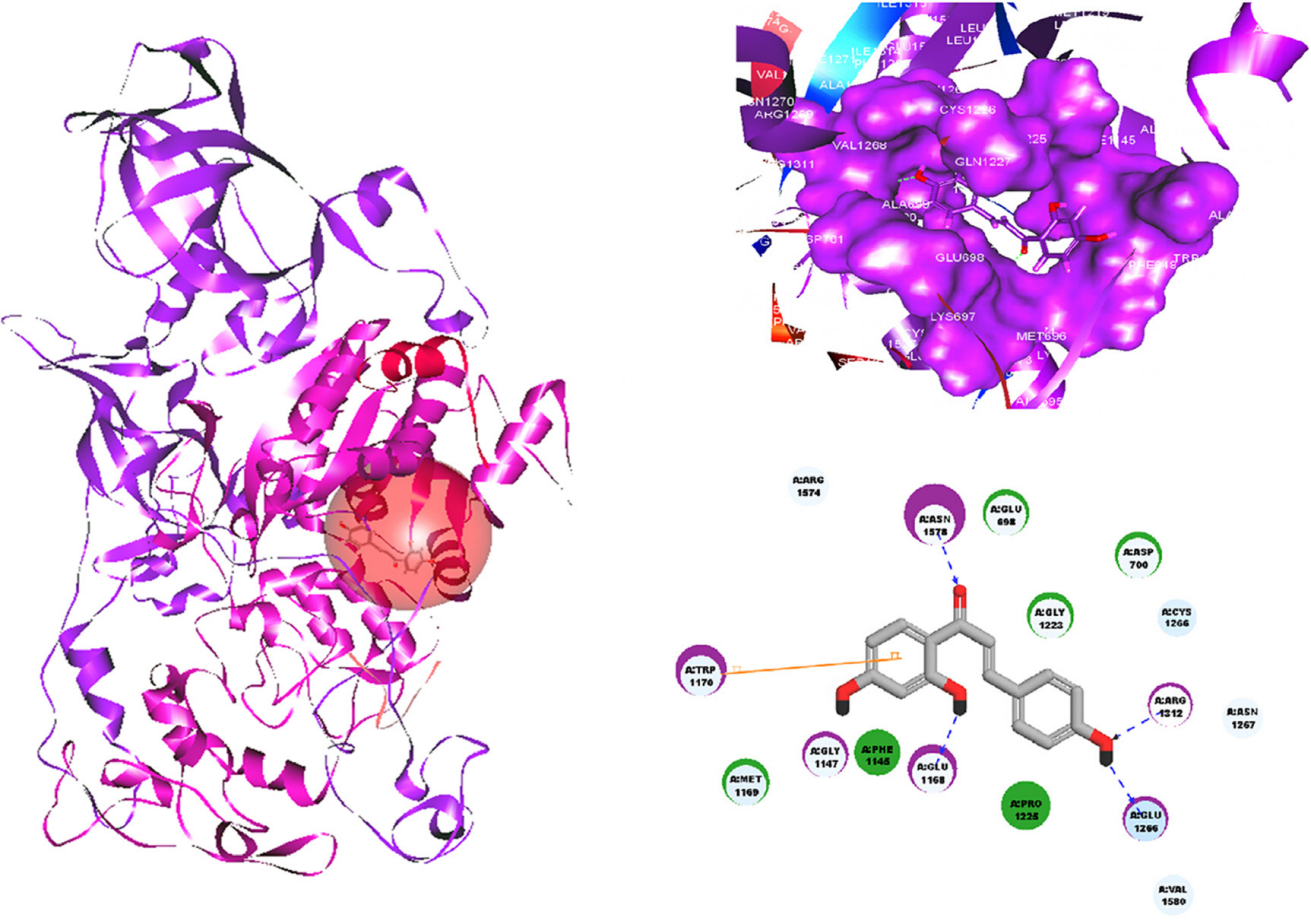
The molecular binding mode of ISL with the catalytic domain within DNMT1 It was predicted by the LigandFit algorithm in Discovery Studio 2.5 using the homology-docking template of 3SWR. It was found that ISL could form stable hydrogen bonds with four active residues including GLU1168, GLU1266, ARG1312 and ASN1578. Meanwhile, ISL could also form a strong π–π interaction with TRP1170. *Blue dashed lines*, hydrogen bonds; *orange line*, π–π interactions.

To further explore the binding mode of ISL with DNMT1 at the molecular level, we conducted a molecular docking analysis of ISL using a homology model 3SWR at the catalytic site of the DNMT1 crystal structure. Using Discovery studio software, we determined that ISL could stably be found at the C-terminal catalytic domain of the catalytic site of DNMT1. In particular, ISL could form stable hydrogen bonds with four active residues including GLU1168, GLU1266, ARG1312 and ASN1578. ISL could also form a strong π–π interactions with TRP1170, which were crucial to the catalytic mechanism of DNMT1 (Figure 9).

## DISCUSSION

Breast cancer is the second leading cause of death among US women, with 39, 620 recorded cancer deaths and 232, 340 new cases of invasive breast cancer in 2013 [36]. The effective prevention of breast cancer has become a global task to further decrease its current incidence ratio and mortality [37–39]. The two major strategies to reduce breast cancer risks include (1) the avoidance of cancer-causing biological, chemical, and physical agents and (2) the habitual consumption of diets high in supplements that protect against cancer [40]. Chemoprevention, the second approach, involves the administration of one or more naturally occurring and/or synthetic agents that can prevent, delay, or even reverse the development of pre-malignant lesions by suppressing the multi-step carcinogenic process [41–42]. In clinical trials, breast cancer chemoprevention has mainly focused on endocrine intervention using selective estrogen receptor modulators (SERMs) and aromatase inhibitors (AIs). Unfortunately, these drugs are active in prevention of hormone responsive lesions only and have poor or no effect in reducing the risk of hormone receptor-negative breast cancer [43–44]. Meanwhile, a number of side effects, such as endometrial cancer and osteoporosis, may be brought forth by SERMs or AIs. Therefore, the development of alternative chemopreventive agents is urgently necessary. Currently, dietary compounds are gaining increased attention for their plentiful resources, multi-target properties, excellent safety profiles and economic value when compared to conventional agents [45–46]. ISL, a representative dietary phenolic compound mainly derived from licorice, has been demonstrated to have potent inhibitory effects on breast cancer cell proliferation, particularly in the estrogen-negative breast cancer cell lines MDA-MB-231 and BT549 [13]. This suggests that ISL has potential to develop as an alternative and novel agent in breast cancer treatment and prevention. Our previous study revealed that it exhibited the greatest inhibitory effects on β-catenin transcription activity among ten types of phytochemical candidates, consequently resulting in its potent anti-cancer effects [13]. Its chemopreventive effects have also been reported recently. Tak *et al*. observed chemopreventive effects in the non-tumorigenic MCF-10A breast cell line [28]. Animal studies found protective effects of ISL against DMBA-induced mammary carcinogenesis, DMBA-induced skin carcinogenesis and azoxy-methane-induced colon carcinogenesis [29, 47–48]. Nevertheless, limited studies have been undertaken to reveal the underlying mechanisms for ISL's chemopreventive potential, particularly in mammary carcinogenesis.

Similar to the above findings, we also observed that ISL was capable of delaying mammary carcinogenesis and cancer metastasis in MMTV-PyMT transgenic mice, accompanied by a significant decrease in the CSC population. Microarray analysis using MMTV-PyMT tumors was conducted to unravel the underlying molecular mechanisms of ISL. The results suggested that the ISL chemopreventive effects might be tightly related to the significant WIF1 augment, which was closely correlated with the regulation of CSC self-renewal. Interestingly, WIF1 inhibition relieved the CSC-limiting effects of ISL, and it was found that ISL stimulated WIF1 expression by promoting its promoter demethylation. It has been reported that aberrant DNA methylation may be one of the earliest events contributing to carcinogenesis [49–50], and our data further demonstrated the central role of WIF1 methylation in breast cancer development and prevention. Restoring WIF1 levels by modifying its DNA methylation patterns has been suggested as an anti-cancer strategy in other malignancies. Tang *et al*. [51] determined that WIF1 could suppress tumor growth *via* G1 arrest in bladder cancer by the down-regulation of SKP2 and c-myc, as well as the up-regulation of p21/WAF1 and p27/Kip1. Rubin *et al*. [52] reported that elevated WIF1 decreased the risks of cancer metastasis in an osteosarcoma mouse model with attenuated expression of MMP-9 and MMP-14. Ramachandran *et al.* [53] found that WIF1 re-expression induced significant apoptosis and G2/M arrest, inhibiting cervical cancer cell proliferation *in vitro* and tumor growth *in vivo*. Most studies elevated WIF1 levels using the common DNA demethylating agent 5-azacytidine (5-Aza) and its derivative 5-aza-20-deoxycytidine (Decitabine), which have been tested in phase I and II trials for many forms of cancer. They have been demonstrated to have significant, although usually transient, improvement in patient survival [54–55]. However, these agents are largely limited due to their clinical toxicity. The major toxicity of these drugs is myelosuppression, and the most commonly reported nonhematologic adverse effect was nausea and vomiting, which was grade 3–4 in approximately 10% of patients [56–57]. Among metastatic lung cancer patients, the toxicity of decitabine occurred in a dose- and schedule-dependent manner, which required a 5–6-week recovery period before the next cycle of therapy [58]. Alternatively, our study suggested using a dietary phytochemical ISL to reverse WIF hypermethylation and suppress mammary tumorigenesis. Previous studies revealed that the minimum lethal dose of ISL was not less than 3000 mg/kg (oral administration) or not less than 1000 mg/kg (intra-peritoneal administration) in 5-week-old mice [59]. In our pilot studies, ISL oral administration posed little toxicity to normal tissues and mammary stem cells in mice, even with chronic administration at 100 mg/kg/d for 3 months, making it desirable as a chemopreventive agent for breast cancer treatment [13]. Collectively, we demonstrated that the dietary compound ISL, at its safe dosages, could effectively reverse the epimutational events, thus suppressing a series of delayed premalignant progressions (including primary/advanced hyperplasia, adenoma) before the onset of mammary malignancy.

DNMTs have been revealed to be central mediators for the aberrant epigenetic regulation [60]. Here, we found that ISL restored WIF1 levels, likely by regulating the enzyme DNMT1, which preferentially methylates hemimethylated DNA over unmethylated DNA. Human DNMT1 is a protein with 1616 amino acids and its structure can be divided into an N-terminal regulatory domain and a C-terminal catalytic domain [61]. DNMT1 disorder was considered to be a significant molecular event to induce carcinogenesis by transferring methyl group from S-adenosylmethionine (SAM) to the fifth position of the cytosine ring [60]. Targeting DNMT1 has become a promising strategy to prevent cancer occurrence. Nowadays, DNMT1 inhibitors could be divided into following categories: (1) trapping DNMT1 through DNA incorporation (such as decitabine, 5-azacytidine and zebularine); (2) blocking the catalytic active site of DNMT1 (such as sinefungin, epigallocatechin gallate (EGCG) and RG108); (3) interrupting the binding site between DNMT1 and DNA (such as procaine and procainamide); (4) inducing DNMT1 degradation (such as decitabine) and (5) suppressing DNMT1 expression (such as antisense MG-98) [62–63].

Based on our findings, ISL might act as both a transcriptional modulator and a chemical inhibitor of DNMT1. It was observed that ISL significantly decreased DNMT1 mRNA and protein expression levels, accompanied with decreased Sp1 levels. Sp1 is a zinc finger transcription factor that preferentially binds to GC-rich motifs of many promoters [64]. Numerous studies reported that SP1 was involved in multiple biological processes, including cell proliferation, cell growth, DNA damage and chromatin remodeling [16, 35, 64]. Clinical studies also indicated that Sp1 expression was aberrantly elevated in various types of cancer, and closely correlated to TNM classification, tumor invasion and lymph node metastasis [65]. With regard to breast cancer, elevated Sp1 was found to regulate thousands of genes that are critically involved in mammary tumorigenesis and metastatic progression, such as vascular endothelial growth factor, urokinase plasminogen activator and its receptor, cyclin D1 and BRCA1 [66]. Recently, Sp1 was also discovered as a transcriptional regulator of DNMT1 [67]. Meanwhile, it was found that Sp1 binding site (5′-GGCGGG-3′) was also exited in the promoter region of WIF1 [68]. Since Sp1 was able to recruit DNMT1 and other methyl CpG-binding proteins following interacting with GC-rich promoters, our results suggested that ISL might enhance WIF1 expression through downregulating Sp1 level, which thereafter inhibited DNMT1 transcription and recruiting to the promoter region of WIF1.

On the other hand, molecular docking analysis indicated that ISL might also potentially act as a chemical inhibitor of DNMT1. Generally, DNMT inhibitors could be divided into nucleoside and non-nucleoside categories [69]. The aforementioned 5-azacytidine, decitabine, and zebularine belong to the nucleoside analogues, which deplete DNMTs by incorporating into DNA and resulting in covalent trapping. The nucleoside analogues have relatively low specificity and are always characterized by substantial cellular and clinical toxicity owing to their direct DNA-destroying properties [70]. Compared to nucleoside analogues, the non-nucleoside inhibitors appear to be less toxic by indirectly blocking DNMT1 activity *via* interfering (through covalent or non-covalent interaction) its catalytic site [71–72]. Yoo *et al.* [73] has applied computational methods, and revealed that many DNMT1 inhibitors could stably dock in the catalytic binding site of DNMT1, including (1) natural products, such as sinefungin, EGCG, curcumin, parthenolide, and mahanine; (2) inhibitors identified from virtual screening, such as RG108 and its analogue RG108–1; and (3) approved drugs for other indications, such as procaine and procainamide. Yoo *et al.* [73] has also proposed that DNMT1 inhibitors commonly share five pharmacophore features at the catalytic site of DNMT1. The best-scoring feature is a negative charge (N) close to the side chains of Ser1230, Gly1231, Lys1535, and Arg1312. The second and third favorable features are a donor site (D) that is near the side chain of Gly1577 and an acceptor site (A) that is close to the side chain of Arg1310 and Arg1312 in the RXR motif. The fourth ranked feature is an aromatic ring (R) that stabilizes the binding conformations of ligands between AdoHcy and Cys1226. The fifth ranked feature is a donor site (D) that is near the side chain of Glu1266, which is a residue implicated in the mechanism of methylation. ISL belongs to the family of non-nucleoside inhibitors. In the present investigation, we predicted the binding mode of ISL with the catalytic domain of DNMT1 using the homology-docking template of the positive DNMT1 catalytic inhibitor sinefungin, and found that ISL matched with the ring (R), donor (D), and acceptor (A) features as stated above, which directly interact with Glu1226 and Arg1312, accompanied by Cys1226 as a surrounding amino acid. However, no direct interaction between ISL and Cys1226 was observed. The results suggested that the inhibitory effects of ISL on DNMT1 activity might largely depend on transcriptional regulation. Further study is still needed to explore the detailed molecular interaction between ISL and DNMT1.

Taken together, our study demonstrated that the dietary compound ISL prevents mammary carcinogenesis by inhibiting breast cancer stem cells through DNMT1-mediated WIF1 demethylation. Our findings not only provide preclinical evidence for supporting ISL as a natural chemopreventive agent but also shed novel light on the development of WIF1 as an epigenetic target for breast cancer prevention.

## MATERIALS AND METHODS

### Chemicals and reagents

ISL was purchased from Alpha Aesar Company (Alfa Aesar, WardHill, MA) with more than 97% purity. The stock solution of ISL was prepared in dimethyl sulfoxide (DMSO) and kept at −20°C. Hochest 33342, 4′, 6-diamidino-2-phenylindole (DAPI), bovine serum albumin (BSA) were obtained from Sigma (Sigma, St. Louis, MO).

### Cell culture

The human breast cancer cell lines MDA-MB-231 and MCF-7 were obtained from the American Type Culture Collection. The cells were cultivated in medium (L-15 for MDA-MB-231; 1640 for MCF-7) supplemented with 10% FBS and 1% penicillin and streptomycin (Gibco Life Technologies, Lofer, Austria) at 37°C in a humidified incubator with or without 5% CO2. The sorted MDA-MB-231 CSCs were subjected to *in vitro* propagation in DMEM/F12 medium supplemented with 1% penicillin and streptomycin, B27 (Invitrogen, Carlsbad, CA), 20 ng/ml hEGF (BD Bioscience, Bedford, MA), 5 μg/ml insulin and 0.4% BSA for the molecular mechanisms study.

### Whole mounting assay

3-week-old female mice were genotyped using PyMT transgene primers that distinguish between the wild type and the PyMT type (Table 1). Mammary glands of PYMT mice were excised, and whole-mounts stained with carmine alum were analyzed as previously described [74]. In particular, the fourth abdominal mammary gland was excised during necropsy, spread on glass slides for 10 min, and fixed in Carnoy's fixative (6 parts 100% ethanol, 3 parts chloroform, and 1 part glacial acetic acid) for 4 h. Subsequently, the tissue was washed in 70% ethanol for 15 min, and the ethanol was changed gradually to distilled water, with a final rinse in distilled water for 5 min. Staining was carried out overnight in carmine alum stain. The tissue was then dehydrated in graded alcohol solutions (70, 95, and 100% for 30 min each) and was cleared in two changes of xylene (30 min each), mounted, and coverslipped using Permount. Whole mounts were recorded using a SPOT FLEX^®^ color digital camera (Diagnostic Instruments, Inc. Sterling Heights, MI).

**Table 1:** Primers used for PCR and sequence analysis

**PyMT**	(F): 5′-GGAAGCAAGTACTTCACAAGGG-3′
	(R):5′-GGAAAGTCACTAGGAGCAGGG-3′
**Smad7**	(F): 5′-ACCCCCATCACCTTAGTCG-3′
	(R):5′-GAAAATCCATTGGGTATCTGGA-3′
**Bmp2**	(F): 5′-AGATCTGTACCGCAGGCACT-3′
	(R):5′-GTTCCTCCACGGCTTCTTC-3′
**Klf4**	(F): 5′-CGGGAAGGGAGAAGACACT-3′
	(R):5′-GAGTTCCTCACGCCAACG-3′
**Abca12**	(F): 5′-CCTGCTAAACCAGACGATCC-3′
	(R): 5′-ACTTGCACAAAGGGGTTCC-3′
**Abca9**	(F): 5′-TGGAAGAATACAGCCTCTCACA-3′
	(R):5′-GCTTCTTCGCCAAAGTCGT-3′
**Car4**	(F): 5′-CAAACCAAGGATCCTAGAAGCA-3′
	(R):5′-GGGGACTGCTGATTCTCCTT-3′
**Fzd4**	(F): 5′-AACTTTCACGCCGCTCAT-3′
	(R):5′-CCGAACAAAGGAAGAACTGC-3′
**Nkd1**	(F): 5′-CACTGTTGGTCGAGGCACT-3′
	(R):5′-CACTTCTAGGGGGAAGTCGTC-3′
**Nkd2**	(F): 5′-GCTATACACCACCGCAGGTC-3′
	(R):5′-GCAGGCTCATTAGCTGGTATG-3′
**Axin2**	(F): 5′-TGGAGGGATGTCCAGTGC-3′
	(R):5′-TGCCAGACATCCTGTGACC-3′
**WIF1**	(F): 5′-GGCAGACACTGCAATAAGAGG-3′
	(R): 5′-TTAAGTGAAGGCGTGTGTCG-3′
**Dkk1**	(F): 5′-CCGGGAACTACTGCAAAAAT-3′
	(R):5′-CCAAGGTTTTCAATGATGCTT-3′
**Ptch2**	(F): 5′-ACAGCTGCTGAGGGCAGA-3′
	(R):5′-CCCGGAAGTGCTCGTACA-3′
**Shh**	(F): 5′-CCAATTACAACCCCGACATC-3′
	(R):5′-GCATTTAACTTGTCTTTGCACCT-3′
**GAPDH**	(F): 5′-CCAATTACAACCCCGACATC-3′
	(R): 5′-GCATTTAACTTGTCTTTGCACCT-3′
**β-catenin**	(F): 5′-GCTTTCAGTTGAGCTGACCA-3′
	(R): 5′-CAAGTCCA AGATCAGCAGTCTC-3′
**Cyclin D1**	(F): 5′-AGGCCGGTGCTGAGTATGTC-3′
	(R): 5′-TGCCTGCTTCACCACCTTCT-3′
**c-Myc**	(F): 5′-GCTGCTTAGACGCTGGATTT-3′
	(R): 5′-TAACGTTGAGGGGCATCG-3′
**survivin**	(F): 5′-CAATTTGCCAAGCTCT GA-3′
	(R): 5′-AGATGGTCGTTTGGCTGAAT-3′
**4-Oct**	(F): 5′-CAATTTGCCAAGCTCCTGA-3′
	(R): 5′-AGATGGTCGTTTGGCTGAAT-3′
**β-actin**	(F): 5′-CCAACCGCGAGAAGATGA-3′
	(R): 5′-CCAGAGGCGTACAG GGATAG-3′
**MSP**	Methylation-specific primers
	(F): 5′-GGGCGTTTTATTGGGCGTAT-3′
	(R): 5′-AAACCAACAATCAACGAAC-3′
	Unmethylation-specific primers
	(F): 5′-GGGTGTTTTATTGGGTGTAT-3′
	(R): 5′-AAACCAACAATCAACAAAAC-3′
**BGS**	(F): 5′-GAGTGATGTTTTAGGGGTTT-3′
	(R): 5′-CCTAAATACCAAAAAACCTAC-3′

### Immunohistochemistry analysis

Paraffin-embedded tumor sample sections (4 μm in thickness) were de-paraffinized in xylene twice for 10 min each and rehydrated using a graded series of ethanol. For histological observation, H&E staining was used. For immunohistochemistry analysis, endogenous peroxidase was inactivated by incubating sections in methanol (with 0.3% hydrogen peroxide) for 30 min at room temperature. Antigen retrieval was performed by heating slides in sodium-citrate buffer (10 mM, pH 6.0) at 94°C for 10 min. The samples were permeabilized in PBS with 0.2% Triton X-100 for 15 min, and nonspecific binding was blocked by 10% normal goat serum for 60 min at room temperature. The slides were then incubated with a 1:100 dilution of primary antibodies WIF1, DNMT1 and DNMT3b (Cell Signaling Technology, Danvers, MA) at 4°C overnight in a moist chamber. After three washes with PBS, the slides were sequentially incubated with biotinylated secondary antibodies for 2 h at room temperature. After three washes with PBS, streptavidin-peroxidase conjugate was added to the samples and incubated for an additional 30 min at room temperature. Finally, the 3, 5-diaminobenzidine (DAB) substrate (Dako A/S, Glostrup, Denmark) was used for color development, followed by counterstaining with Mayer's hematoxylin. Antigen-positive cells were counted in six fields per tumor sample. The results were expressed as the average ± SD of tumors per group.

### Flow cytometry analysis

Primary mouse mammary cells or breast cancer cell lines (MDA-MB-231 and MCF-7) were washed with PBS and then harvested with trypsin. The detached cells were washed with PBS containing 1% FBS (wash buffer), and resuspended in the wash buffer (10^6^ cells/100 μl). For SP analysis, cells were stained with Hoechst 33342 (5 μg/ml) in medium at 37°C for 2 h, and resuspended in ice-cold PBS containing 2 μg/ml propidium iodide (PI). For ALDEFLUOR assay, the experiment was performed using aldehyde dehydrogenase-based cell detection kit (Stem Cell Technologies, Grenoble, France) as described previously [75]. Briefly, 10^6^ cells were suspended in Aldefluor^®^ assay buffer containing ALDH substrate (Bodipy-Aminoacetaldehyde) and incubated for 45 min at 37°C. As a reference control, the cells were suspended in buffer containing Aldefluor^®^ substrate in the presence of diethylaminobenzaldehyde (DEAB), a specific ALDH1 enzyme inhibitor. The brightly fluorescent ALDH1-expressing cells (ALDH1^high^) were detected by a 488 nm blue laser. For the *in vitro* stem cell analysis/sorting, cells were incubated with combinations of fluorescence-conjugated monoclonal antibodies obtained from BD Biosciences (San Diego, CA, USA) against human CD44-FITC and CD24-PE at 4°C in the dark for 40 min and then washed once with PBS. FITC- or PE-labeled isotype IgG1 was used as the negative control. For cell cycle analysis, cells were fixed with ice-cold 70% ethanol at −20°C overnight. The next day, the cells were washed with PBS, stained with 50 mg/mL propidium iodide (Sigma, St. Louis, MO), and dissolved in 100 mg/L RNase A (Sigma, St. Louis, MO). Flow cytometry analysis was conducted with FACSAria SORP (BD Biosciences, San Jose, CA), and analyzed by FlowJo or Modifit LT software.

### Microarray analysis

Total RNA was harvested from mammary tumor tissues of vehicle or ISL-treated MMTV-PyMT mice, extracted using TRIzol reagent (Invitrogen, Carlsbad, CA), and further purified with RNeasy kits (Qiagen, Chatsworth, CA). Microarray analysis was performed with the Affymetrix GeneChip^®^ Mouse Gene 2.0 ST array (Affymetrix, Santa Clara, CA) by the Centre for Genomic Sciences of HKU. Triplicate independent RNA preparations of tumors from vehicle and ISL-treated groups were compared. The results were analyzed by GeneSpring12.6 with normalization of the raw gene expression data, quality control checks, and subsequent analyses. Genes that changed by at least 2.0-fold between groups and with a *P*-value of less than 0.05 were finally defined as target genes.

### Real-time PCR analysis

Total RNA from primary mammary tumor tissues or MDA-MB-231 CSCs was extracted using TRIzol reagent (Invitrogen, Carlsbad, CA), and reverse transcription was carried out using a first strand cDNA synthesis kit (Roche Diagnostics, IN) according to the manufacturer's instructions. Real-time PCR analysis was performed using a SYBR Green kit (Roche Diagnostics, IN) on a Roche LightCycler 480 detector. The reactions were incubated in a 96-well plate at 95°C for 10 min followed by 40 cycles of 95°C for 10 s, 55°C for 30 s and 72°C for 1 min. The designed primers were listed in Table 1. The Ct value was measured during the exponential amplification phase. The relative expression level (defined as fold change) of the target genes was given by 2^−ΔΔCt^ and normalized to the internal control.

### Western blot analysis

To determine the protein concentration, cells were lysed in RIPA buffer (Sigma, St. Louis, MO) containing a protease inhibitor mixture (Roche Diagnostics, IN). The protein concentration was measured with the bicinchoninic acid assay (Thermo Fisher Scientific, Bonn, Germany). Quantified protein lysates (15 μg) were subjected to sodium dodecyl sulfate polyacrylamide gel electrophoresis (SDS-PAGE) and resolved on 12% polyacrylamide gels. The proteins were then transferred onto a PVDF membrane (GE Healthcare, Freiburg, Germany). The membrane was probed with primary antibodies, including WIF1, β-catenin, P-β-catenin (Ser33/37/Thr41), Akt, P-Akt (Ser473), GSK3β, P-GSK3β (Ser9), DNMT1, DNMT3b, lamin B and β-actin (Cell Signaling Technology, Danvers, MA) at 4°C overnight. After three washes with Tris-buffered saline with 0.05% Tween-20, the membrane was incubated with secondary anti-rabbit or anti-mouse antibodies (Cell Signaling Technology, Danvers, MA) for 2 h at room temperature. The signals were visualized using the ECL Advance reagent (GE Healthcare) and quantified using Quantity One software.

### Immunofluorescence analysis

The untreated and ISL-treated MDA-MB-231 and MCF-7 breast cancer cells were fixed in 4% paraformaldehyde for 10 min and permeabilized with 0.2% triton X-100. After blocking in 10% goat serum for 1 h, the slide was incubated with primary WIF1 antibody (Santa Cruz, CA) overnight at 4°C, followed by secondary fluorescence-labeled antibodies (Santa Cruz, CA) for 2 h at room temperature. Finally, the samples were incubated with DAPI for nuclear staining and the signal was detected with a confocal microscope.

### Mammosphere formation assays

The sorted breast CSCs from MDA-MB-231 and MCF-7 were plated and cultured in 24-well ultralow attachment plates at a density of 1000 cells per well. The plating medium for mammosphere formation consisted of DMEM/F12 medium supplemented with B27, 20 ng/ml hEGF, 5 μg/ml insulin, 0.4% BSA and 1% penicillin and streptomycin. To examine the effects of ISL on the formation of mammospheres, the 3^rd^ day mammospheres were treated with varying concentrations of ISL. The plating medium was refreshed every 3 days in the absence of additional ISL treatment and the appearance of primary spheres was evaluated after 5 days. To assess the effects of ISL over the secondary and the third passage, mammospheres from the previous plating were collected and dissociated into single-cell suspensions with 0.05% trypsin, filtered using a 40-μm sieve and re-plated in ultra-low attachment plates. The number and size of the resulting mammospheres were observed with no additional treatment. Three independent experiments were conducted.

### MSP and BGS analysis

MDA-MB-231 and MCF-7 cells were treated with or without varying concentrations of ISL for varied time intervals. After treatment, the genomic DNA was extracted using the Genomic DNA Mini Kit for Cultured Cells (Geneaid Biotech Ltd, Sijhih City, Taiwan) according to the manufacturer's instructions. Approximately 0.5 μg of genomic DNA was bisulfite-modified using the EZ DNA Methylation Kit (Zymo Research, Orange, CA). The WIF1 promoter region has been identified and described previously [76]. The methylation sites for MSP and BGS at WIF1 prompter were also validated by the studies of Mazieres *et al*. [77] and Yang *et al.* [78]. For MSP analysis, bisulfite-treated genomic DNA was amplified using either a methylation-specific or an unmethylation-specific primer set targeting the WIF1 promoter region sequences from −488 to −290. The designed methylation- and unmethylation-specific primers were listed in Table 1. The PCR was carried out under the following conditions: one cycle of 95°C for 10 min, followed by 35 cycles of denaturing at 94°C for 1 min, annealing at 60°C for 50 s and extension at 72°C for 50 s, which was followed by the final extension at 72°C for 10 min. The PCR products were analyzed by electrophoresis on a 2% agarose gel and samples were visualized with a UV imaging system. For BGS sequencing analysis, bisulfite-treated genomic DNA was amplified using primers designed to amplify nucleotides from −555 to −140 of the WIF1 promoter region. The designed primers were listed in Table 1. The PCR was carried out under the following conditions: one cycle of 94°C for 2 min, followed by 35 cycles of denaturing at 94°C for 15 s, annealing at 55°C for 30 s and extension at 68°C for 40 s, which was followed by the final extension at 72°C for 15 min. The PCR products were cloned into the pCRTM4-TOPO^®^ Vector and transformed into competent E. coli DH5a using the TOPO^®^ TA cloning^®^ kit (Invitrogen, Carlsbad, CA). Positive clones were selected for sequencing using M13 primer in the Genomic Centre of the University of Hong Kong. The DNA methylation analysis was conducted with software BiQ Analyzer [79].

### DNMT1 siRNA construction and transfection

The siRNAs targeting DNMT1 or their scrambled siRNAs were purchased from Invitrogen (Carlsbad, CA, USA) and transfected into breast cancer cells MDA-MB-231 using X-tremeGENE siRNA transfection reagent (Roche Diagnostics, IN) according to the manufacturer's instructions.

### Molecular docking

The LigandFit algorithm in Discovery Studio 2.5 was used in the molecular docking study. The LigandFit algorithm employed a Monte Carlo conformational search procedure to generate ligand docking conformations. The chemical structure of ISL was drawn by Chemoffice 2002 (CambridgeSoft, Cambridge, MA). The crystal structure of the DNMT1 catalytic structure was obtained from the Protein Data Bank (http://www.rcsb.org/pdb/) with the ID of 3SWR. The water molecules in DNMT1 were removed. For docking purposes, the catalytic site of the Cys 1226 domain within the DNMT1 catalytic structure was defined as the ligand-binding site and ISL was docked into DNMT1 with the correct parameter settings. The potentials to form hydrogen bonds and π-π interactions within the active site were calculated. Docking with no output pose was considered a failure. Accuracy testing was performed by calculating the root mean square deviation (RMSD) after re-docking the internal ligand with the algorithm into the crystal structure of the DNMT1 catalytic structure.

### Statistical analysis

The data were shown as the mean ± SD. A two-tailed Student's *t*-test or one-way analysis of variance was used to determine the significance of the data between groups. Statistical significance was reached when the *P* value < 0.05.

## SUPPLEMENTARY FIGURE



## Abbreviations

CSC: cancer stem cell
MSP: methylation-specific polymerase chain reaction
BGS: bisulfite genomic sequencing
LEF/TCF: lymphocyte enhancer factor/T cell factor family of transcription factors
WIF1: Wnt inhibitory factor 1
ISL: Isoliquiritigenin
EGCG: Epigallocatechin-3-gallate
COX-2: Cyclooxygenase-2
MMTV-PyMT: mouse mammary tumor virus promoter-driven polyoma middle T oncoprotein
H&E: hematoxylin and eosin
SP: side population
ALDH: Aldehyde dehydrogenase
DEAB: diethylaminobenzaldehude
CM: conditional medium
DNMTs: DNA methyltransferases
SERMs: selective estrogen receptor modulators
AIs: aromatase inhibitors
5-Aza: 5-azacytidine
Decitabine: 5-aza-20 deoxycytidine
SAM: S-Adenosyl-L-methionine
DMSO: dimethyl sulfoxide
DAPI: 4′,6-diamidino-2-phenylindole
BSA: bovine serum albumin
PI: propidium iodide
SDS-PAGE: sodium dodecyl sulfate polyacrylamide gel electrophoresis
RMSD: root mean square deviation

## References

[1] Jemal A, Bray F, Center MM, Ferlay J, Ward E, Forman D (2011). Global cancer statistics. CA Cancer J Clin.

[2] Al-Hajj M, Wicha MS, Benito-Hernandez A, Morrison SJ, Clarke MF (2003). Prospective identification of tumorigenic breast cancer cells. Proc Natl Acad Sci U S A.

[3] Subramaniam D, Ramalingam S, Houchen CW, Anant S (2010). Cancer stem cells: a novel paradigm for cancer prevention and treatment. Mini Rev Med Chem.

[4] Li F, Tiede B, Massague J, Kang Y (2007). Beyond tumorigenesis: cancer stem cells in metastasis. Cell Res.

[5] Curtin JC, Lorenzi MV (2010). Drug discovery approaches to target Wnt signaling in cancer stem cells. Oncotarget.

[6] Nusse R, Varmus HE (1982). Many tumors induced by the mouse mammary tumor virus contain a provirus integrated in the same region of the host genome. Cell.

[7] Li Y, Hively WP, Varmus HE (2000). Use of MMTV-Wnt-1 transgenic mice for studying the genetic basis of breast cancer. Oncogene.

[8] Huguet EL, McMahon JA, McMahon AP, Bicknell R, Harris AL (1994). Differential expression of human Wnt genes 2, 3, 4, and 7B in human breast cell lines and normal and disease states of human breast tissue. Cancer Res.

[9] Polakis P (2000). Wnt signaling and cancer. Genes Dev.

[10] Khramtsov AI, Khramtsova GF, Tretiakova M, Huo D, Olopade OI, Goss KH (2010). Wnt/β-catenin pathway activation is enriched in basal-like breast cancers and predicts poor outcome. Am J Pathol.

[11] Su Y, Fu C, Ishikawa S, Stella A, Kojima M, Shitoh K, Schreiber EM, Day BW, Liu B (2008). APC is essential for targeting phosphorylated β-catenin to the SCFβ-TrCP ubiquitin ligase. Mol Cell.

[12] He JP, Hao Y, Wang XL, Yang XJ, Shao JF, Guo FJ, Feng JX (2014). Review of the molecular pathogenesis of osteosarcoma. Asian Pac J Cancer Prev.

[13] Wang N, Wang ZY, Peng C, You JS, Shen JG, Han SW, Chen JP (2014). Dietary compound isoliquiritigenin targets GRP78 to chemosensitize breast cancer stem cells via β-catenin/ABCG2 signaling. Carcinogenesis.

[14] Kawano Y (2003). J Cell Sci.

[15] Suzuki M, Shigematsu H, Nakajima T, Kubo R, Motohashi S, Sekine Y, Shibuya K, Iizasa T, Hiroshima K, Nakatani Y, Gazdar AF, Fujisawa T (2007). Synchronous alterations of Wnt and epidermal growth factor receptor signaling pathways through aberrant methylation and mutation in non small cell lung cancer. Clin Cancer Res.

[16] Ai L, Tao Q, Zhong S, Fields CR, Kim WJ, Lee MW, Cui Y, Brown KD, Robertson KD (2006). Inactivation of Wnt inhibitory factor-1 (WIF1) expression by epigenetic silencing is a common event in breast cancer. Carcinogenesis.

[17] Veeck J, Wild PJ, Fuchs T, Schuffler PJ, Hartmann A, Knuchel R, Dahl E (2009). Prognostic relevance of Wnt-inhibitory factor-1 (WIF1) and Dickkopf-3 (DKK3) promoter methylation in human breast cancer. BMC Cancer.

[18] Anastas JN, Moon RT (2013). WNT signalling pathways as therapeutic targets in cancer. Nat Rev Cancer.

[19] Landis-Piwowar KR, Iyer NR (2014). Cancer chemoprevention: current state of the art. Cancer Growth Metastasis.

[20] Li Y, Wicha MS, Schwartz SJ, Sun D (2011). Implications of cancer stem cell theory for cancer chemoprevention by natural dietary compounds. J Nutr Biochem.

[21] Park CH, Chang JY, Hahm ER, Park S, Kim HK, Yang CH (2005). Quercetin, a potent inhibitor against β-catenin/Tcf signaling in SW480 colon cancer cells. Biochem Biophys Res Commun.

[22] Kim J, Zhang X, Rieger-Christ KM, Summerhayes IC, Wazer DE, Paulson KE, Yee AS (2006). Suppression of Wnt signaling by the green tea compound (−)-epigallocatechin 3-gallate (EGCG) in invasive breast cancer cells. Requirement of the transcriptional repressor HBP1. J Biol Chem.

[23] Lee YM, Jeong GS, Lim HD, An RB, Kim YC, Kim EC (2010). Isoliquiritigenin 2′-methyl ether induces growth inhibition and apoptosis in oral cancer cells via heme oxygenase-1. Toxicol In Vitro.

[24] Park I, Park KK, Park JH, Chung WY (2009). Isoliquiritigenin induces G2 and M phase arrest by inducing DNA damage and by inhibiting the metaphase/anaphase transition. Cancer Lett.

[25] Wang ZY, Wang N, Han SW, Wang D, Mo S, Yu L, Huang H, Tsui K, Shen JG, Chen JP (2013). Dietary compound isoliquiritigenin inhibits breast cancer neoangiogenesis via VEGF/VEGFR-2 signaling pathway. PLoS One.

[26] Kwon GT, Cho HJ, Chung WY, Park KK, Moon A, Park JH (2009). Isoliquiritigenin inhibits migration and invasion of prostate cancer cells: possible mediation by decreased JNK/AP-1 signaling. J Nutr Biochem.

[27] Chen G, Hu X, Zhang W, Xu N, Wang FQ, Jia J WF, Sun ZJ, Zhao YF (2012). Mammalian target of rapamycin regulates isoliquiritigenin-induced autophagic and apoptotic cell death in adenoid cystic carcinoma cells. Apoptosis.

[28] Lau GT, Ye L, Leung LK (2010). The licorice flavonoid isoliquiritigenin suppresses phorbol ester-induced cyclooxygenase-2 expression in the non-tumorigenic MCF-10A breast cell line. Planta Med.

[29] Cuendet M, Guo J, Luo Y, Chen S, Oteham CP, Moon RC, van Breemen RB, Marler LE, Pezzuto JM (2010). Cancer chemopreventive activity and metabolism of isoliquiritigenin, a compound found in licorice. Cancer Prev Res (Phila).

[30] Wang C, Schwab LP, Fan M, Seagroves TN, Buolamwini JK (2013). Chemoprevention activity of dipyridamole in the MMTV-PyMT transgenic mouse model of breast cancer. Cancer Prev Res (Phila).

[31] Franci C, Zhou J, Jiang Z, Modrusan Z, Good Z, Jackson E, Kouros-Mehr H (2013). Biomarkers of residual disease, disseminated tumor cells, and metastases in the MMTV-PyMT breast cancer model. PLoS One.

[32] Lin EY, Jones JG, Li P, Zhu L, Whitney KD, Muller WJ, Pollard JW (2003). Progression to malignancy in the polyoma middle T oncoprotein mouse breast cancer model provides a reliable model for human diseases. Am J Pathol.

[33] Tu SM (2013). Cancer: a “stem-cell” disease?. Cancer Cell Int.

[34] Ginestier C, Hur MH, Charafe-Jauffret E, Monville F, Dutcher J, Brown M, Jacquemier J, Viens P, Kleer CG, Liu S, Schott A, Hayes D, Birnbaum D, Wicha MS, Dontu G (2007). ALDH1 is a marker of normal and malignant human mammary stem cells and a predictor of poor clinical outcome. Cell Stem Cell.

[35] Liu J, Lam JB, Chow KH, Xu A, Lam KS, Moon RT, Wang Y (2008). Adiponectin stimulates Wnt inhibitory factor-1 expression through epigenetic regulations involving the transcription factor specificity protein 1. Carcinogenesis.

[36] DeSantis C, Ma J, Bryan L, Jemal A (2014). Breast cancer statistics, 2013. CA Cancer J Clin.

[37] Weinberg RA (1996). How cancer arises. Sci Am.

[38] Lori J, Sharad G, Paul F, Jeffrey W, Melanie R, Shari BG (2014). Breast Cancer Overview: Risk Factors, Screening, Genetic Testing, and Prevention. Cancer Management.

[39] Tsao AS, Kim ES, Hong WK (2004). Chemoprevention of cancer. CA Cancer J Clin.

[40] Amin AR, Kucuk O, Khuri FR, Shin DM (2009). Perspectives for cancer prevention with natural compounds. J Clin Oncol.

[41] Kelloff G (2000). Perspectives on cancer chemoprevention research and drug development. Adv Cancer Res.

[42] Crowell JA (2005). The chemopreventive agent development research program in the Division of Cancer Prevention of the US National Cancer Institute: an overview”. Eur J Cancer.

[43] Cazzaniga M, Bonanni B (2012). Breast cancer chemoprevention: old and new approaches. J Biomed Biotechnol.

[44] den Hollander P, Savage MI, Brown PH (2013). Targeted therapy for breast cancer prevention. Front Oncol.

[45] El-Bayoumy K, Chung FL, Richie JJr, Reddy BS, Cohen L, Weisburger J, Wynder EL (1997). Dietary control of cancer. Proc Soc Exp Biol Med.

[46] Yang CS, Feng Q (2014). Chemo/Dietary prevention of cancer: perspectives in China. J Biomed Res.

[47] Yamamoto S, Aizu E, Jiang H, Nakadate T, Kiyoto I, Wang JC, Kato R (1991). The potent anti-tumor-promoting agent isoliquiritigenin. Carcinogenesis.

[48] Baba M, Asano R, Takigami I, Takahashi T, Ohmura M, Okada Y, Sugimoto H, Arika T, Nishino H, Okuyama T (2002). Studies on cancer chemoprevention by traditional folk medicines XXV. Inhibitory effect of isoliquiritigenin on azoxymethane-induced murine colon aberrant crypt focus formation and carcinogenesis. Biol Pharm Bull.

[49] Pogribny IP (2010). Epigenetic events in tumorigenesis: putting the pieces together. Exp Oncol.

[50] Baylin SB, Herman JG (2000). DNA hypermethylation in tumorigenesis: epigenetics joins genetics”. Trends Genet.

[51] Tang Y, Simoneau AR, Liao WX, Yi G, Hope C, Liu F, Li S, Xie J, Holcombe RF, Jurnak FA, Mercola D, Hoang BH, Zi X (2009). WIF1, a Wnt pathway inhibitor, regulates SKP2 and c-myc expression leading to G1 arrest and growth inhibition of human invasive urinary bladder cancer cells”. Mol Cancer Ther.

[52] Rubin EM, Guo Y, Tu K, Xie J, Zi X, Hoang BH (2010). Wnt inhibitory factor 1 decreases tumorigenesis and metastasis in osteosarcoma. Mol Cancer Ther.

[53] Ramachandran I, Thavathiru E, Ramalingam S, Natarajan G, Mills WK, Benbrook DM, Zuna R, Lightfoot S, Reis A, Anant S, Queimado L (2012). Wnt inhibitory factor 1 induces apoptosis and inhibits cervical cancer growth, invasion and angiogenesis *in vivo*. Oncogene.

[54] Weiss AJ, Stambaugh JE, Mastrangelo MJ, Laucius JF, Bellet RE (1972). Phase I study of 5-azacytidine (NSC-102816). Cancer Chemother Rep.

[55] Weiss AJ, Metter GE, Nealon TF, Keanan JP, Ramirez G, Swaiminathan A, Fletcher WS, Moss SE, Manthei RW (1977). Phase II study of 5-azacytidine in solid tumors. Cancer Treat Rep.

[56] Vesely J, Cihak A (1977). Possibilities for the clinical use of 5-azacytidine. Vopr Onkol.

[57] Saiki JH, McCredie KB, Vietti TJ, Hewlett JS, Morrison FS, Costanzi JJ, Stuckey WJ, Whitecar J, Hoogstraten B (1978). 5-azacytidine in acute leukemia. Cancer.

[58] Momparler RL, Bouffard DY, Momparler LF, Dionne J, Belanger K, Ayoub J (1997). Pilot phase I-II study on 5-aza-2′-deoxycytidine (Decitabine) in patients with metastatic lung cancer”. Anticancer Drugs.

[59] Baba M, Asano R, Takigami I, Takahashi T, Ohmura M, Okada Y, Sugimoto H, Arika T, Nishino H, Okuyama T (2002). Studies on cancer chemoprevention by traditional folk medicines XXV. Inhibitory effect of isoliquiritigenin on azoxymethane-induced murine colon aberrant crypt focus formation and carcinogenesis. Biol Pharm Bull.

[60] Joska TM, Zaman R (2014). Belden WJ. Regulated DNA methylation and the circadian clock: implications in cancer. Biology (Basel).

[61] Cheng X, Blumenthal RM (2008). Mammalian DNA methyltransferases: a structural perspective. Structure.

[62] Liu Z, Xie Z, Jones W, Pavlovicz RE, Liu S, Yu J, Li PK, Lin J, Fuchs JR, Marcucci G, Li C, Chan KK (2009). Curcumin is a potent DNA hypomethylation agent. Bioorg Med Chem Lett.

[63] Yu J, Peng Y, Wu LC, Xie Z, Deng Y, Hughes T, He S, Mo X, Chiu M, Wang QE, He X, Liu S, Grever MR, Chan KK, Liu Z (2013). Curcumin down-regulates DNA methyltransferase 1 and plays an anti-leukemic role in acute myeloid leukemia. PLoS One.

[64] Safe S1, Abdelrahim M (2005). Sp transcription factor family and its role in cancer. Eur J Cancer.

[65] Wang XB, Peng WQ, Yi ZJ, Zhu SL, Gan QH (2007). Expression and prognostic value of transcriptional factor sp1 in breast cancer. Ai Zheng.

[66] Zannetti A1, Del Vecchio S, Romanelli A, Scala S, Saviano M, Cali' G, Stoppelli MP, Pedone C, Salvatore M (2005). Inhibition of Sp1 activity by a decoy PNA-DNA chimera prevents urokinase receptor expression and migration of breast cancer cells. Biochem Pharmacol.

[67] Maor S, Papa MZ, Yarden RI, Friedman E, Lerenthal Y, Lee SW, Mayer D, Werner H (2007). Insulin-like growth factor-I controls BRCA1 gene expression through activation of transcription factor Sp1. Horm Metab Res.

[68] Chang WC, Hung JJ (2012). Functional role of post-translational modifications of Sp1 in tumorigenesis. J Biomed Sci.

[69] Lyko F, Brown R (2005). DNA methyltransferase inhibitors and the development of epigenetic cancer therapies. J Natl Cancer Inst.

[70] Christman JK (2002). 5-Azacytidine and 5-aza-2′-deoxycytidine as inhibitors of DNA methylation: mechanistic studies and their implications for cancer therapy. Oncogene.

[71] Pan MH, Chiou YS, Chen LH, Ho CT (2014). Breast cancer chemoprevention by dietary natural phenolic compounds: specific epigenetic-related molecular targets. Mol Nutr Food Res.

[72] Chikan NA, Bhavaniprasad V, Anbarasu K, Shabir N, Patel TN (2013). From natural products to drugs for epimutation computer-aided drug design”. Appl Biochem Biotechnol.

[73] Yoo J, Kim JH, Robertson KD, Medina-Franco JL (2012). Molecular modeling of inhibitors of human DNA methyltransferase with a crystal structure: discovery of a novel DNMT1 inhibitor. Adv Protein Chem Struct Biol.

[74] Nagy T, Wei H, Shen TL, Peng X, Liang CC, Gan B, Guan JL (2007). Mammary epithelial-specific deletion of the focal adhesion kinase gene leads to severe lobulo-alveolar hypoplasia and secretory immaturity of the murine mammary gland. J Biol Chem.

[75] Lohberger B, Rinner B, Stuendl N, Absenger M, Liegl-Atzwanger B, Walzer SM, Windhager R, Leithner A (2012). Aldehyde dehydrogenase 1, a potential marker for cancer stem cells in human sarcoma. PLoS One.

[76] Li LC, Dahiya R (2002). MethPrimer: designing primers for methylation PCRs. Bioinformatics.

[77] Mazieres J, He B, You L, Xu Z, Lee AY, Mikami I, Reguart N, Rosell R, McCormick F, Jablons DM (2004). Wnt inhibitory factor-1 is silenced by promoter hypermethylation in human lung cancer. Cancer Res.

[78] Yang Z, Wang Y, Fang J, Chen F, Liu J, Wu J, Song T, Zeng F, Rao Y (2010). Downregulation of WIF-1 by hypermethylation in astrocytomas. Acta Biochim Biophys Sin.

[79] Bock C, Reither S, Mikeska T, Paulsen M, Walter J, Lengauer T (2005). BiQ Analyzer: visualization and quality control for DNA methylation data from bisulfite sequencing. Bioinformatics.

