# Supplementary material for: Dietary compound isoliquiritigenin prevents mammary carcinogenesis by inhibiting breast cancer stem cells through WIF1 demethylation
Source: Oncotarget. 2015 Mar 26;6(12):9854–76. doi: 10.18632/oncotarget.3396 (PMC4496402; doi:10.18632/oncotarget.3396)
Supplement: Supplementary file 1 [file oncotarget-06-9854-s001.pdf]

SUPPLEMENTARY FIGURE

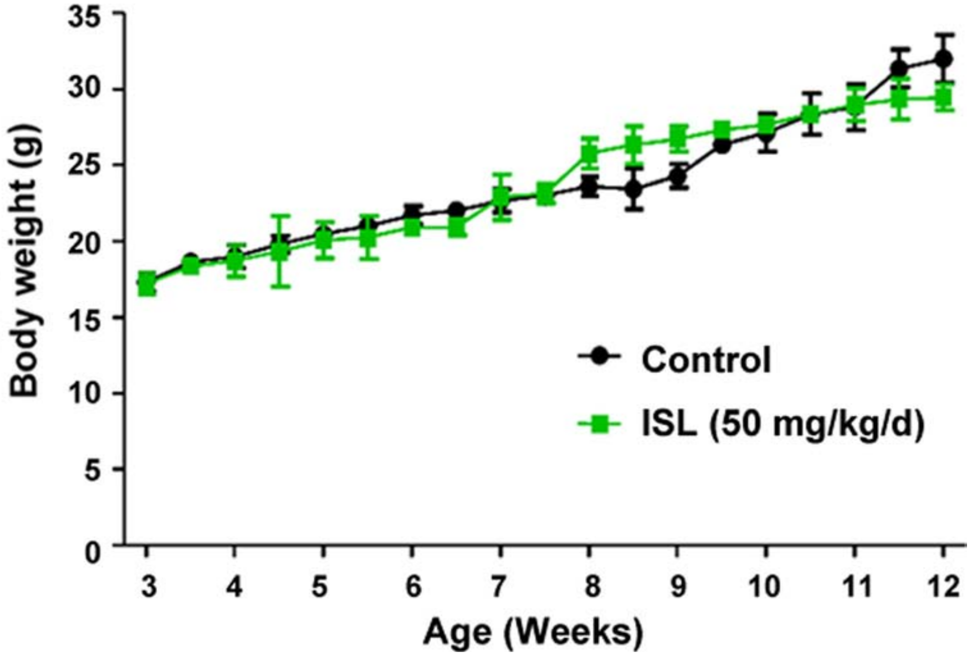

Supplementary Figure S1: ISL brought little influences on mice body weight.
